# Sex-specific differences in the relationship between the atherogenic index and hypertension in middle-aged and elderly Chinese

**DOI:** 10.3389/fendo.2025.1574125

**Published:** 2025-06-18

**Authors:** Zhengfeng Zhang, Jingyu Wang, Leilei Kong, Congcong Li, Wanwan Bao, Huaijun Tu, Jian Li

**Affiliations:** ^1^ The Second Affiliated Hospital, Jiangxi Medical College, Nanchang University, Nanchang, Jiangxi, China; ^2^ The Department of Geratology, The Second Affiliated Hospital of Nanchang University, Nanchang, Jiangxi, China

**Keywords:** atherogenic index of plasma, sex-specific differences, female, hypertension, pre-hypertension

## Abstract

**Background:**

Despite the already comprehensive epidemiological evidence concerning pre-hypertension, high-normal blood pressure, and hypertension, the influence of gender differences within this context remains inadequately explored. The present study endeavors to meticulously examine the specific impact of the plasma atherogenic index (AIP) on pre-hypertension and hypertension, and ascertain whether there exist significant sex-specific differences in this regard.

**Methods:**

This population-based study employed a multi-wave cohort design encompassing 8255 middle-aged and elderly participants (cross-sectional phase) and longitudinal follow-ups in 2015 (n=8092) and 2018 (n=7022). Participants were stratified into normotensive (n=3175 in cross-sectional, n=2415 in 2015 longitudinal cohort study, 1868 in 2018 longitudinal cohort study) and prehypertensive/hypertensive groups (n=5080 (61.5%) in cross-sectional study, n=5677(70.2%) in longitudinal study of 2015, n=5336(76.0%) in 2018). The plasma atherogenic index=log10(triglycerides/high-density lipoprotein)[triglycerides (mg/dL)/HDL-C (mg/dL)]) was quantified enzymatically. Multivariable-adjusted logistic regression models with restricted cubic splines were implemented to evaluate nonlinear associations between AIP and blood pressure status, adjusting for age, sex, BMI, smoking, and lipid-lowering therapy. Sensitivity analyses included multiple imputation for missing covariates and sex-stratified effect modification testing.

**Results:**

This epidemiological investigation revealed population prevalences of 34.3% for pre-hypertension and 27.2% for hypertension. Both cross-sectional and longitudinal analyses demonstrated a significant positive association between AIP index and blood pressure dysregulation. Adjusted logistic regression models showed that elevated AIP corresponded to increased risks of pre-hypertension/hypertension, with cross-sectional analyses yielding an odds ratio (OR) of 1.69 (95% CI:1.38 to 2.07, P<0.001). Longitudinal cohorts of 2015 and 2018 exhibited persistent temporal trends: OR=1.38 (95% CI:1.13 to 1.67, P=0.012) in 2015 and OR=1.41 (95% CI:1.20 to 1.65, P<0.001) in 2018. Sex-stratified analyses revealed markedly stronger associations in females, where each AIP unit increase conferred a 1.79-fold cross-sectional risk elevation (OR: 1.79, 95% CI:1.35 to 2.38, P < 0.001), rising to 1.49-fold (2015 cohort: OR: 1.49, 95% CI: 1.14 to 1.95, P=0.003) and 1.64-fold (2018 cohort: OR: 1.64, 95% CI:1.31 to 2.06, P<0.001) in longitudinal assessments. Conversely, males exhibited attenuated associations (cross-sectional OR: 1.30; 95% CI:1.12 to 1.79, P=0.006; 2015 longitudinal OR: 1.26, 95% CI:1.12 to 1.66), with nonsignificant effects in the 2018 follow-up (OR: 0.87, 95% CI:0.57 to 1.31). A significant gender-AIP interaction (P<0.001) underscored sex-specific metabolic susceptibility to atherogenic lipid profiles.

**Conclusion:**

This study identified a significant positive association between elevated atherogenic index of plasma levels and blood pressure dysregulation. Both cross-sectional and longitudinal analyses consistently demonstrated a dose-response relationship, with higher AIP levels associated with increased risk. Stratified analyses by sex revealed that the association between elevated AIP and the incidence of pre-hypertension and hypertension was significantly stronger in women.

## 1 Introduction

Hypertension, or elevated blood pressure (HBP), stands as a prevalent and significant cardiovascular concern. Alongside diabetes and abnormal blood lipid levels, it ranks among the top three chronic diseases in our country, with hypertension topping the list, affecting over 270 million individuals (1). According to data from the Noncommunicable Disease Risk Factor Collaboration (NCDRisC), the global population of individuals with hypertension surpassed one billion in 2019, marking a doubling since 1990 (2, 3). This chronic non-communicable disease profoundly and persistently impacts the daily lives of the populace (4).

The initial stage of hypertension, known as high-normal blood pressure, refers to blood pressure levels that exceed the normal range but fall below the diagnostic threshold for hypertension. This phase is intricately tied to the development of hypertension and the risk of associated complications, underscoring the importance of recognizing and addressing this preliminary stage (5). Notably, numerous epidemiological studies have highlighted that an increase in blood pressure to a pre-hypertensive state can adversely affect multiple vital organs and tissues, including the heart, cerebrovascular system, kidneys, and retina. This elevation is associated with the exacerbation of conditions such as coronary atherosclerotic heart disease, myocardial infarction, kidney damage, stroke, and advanced cognitive decline (6).Compared to individuals with blood pressure within the normal range, those in the pre-hypertension stage exhibit a notably elevated risk of developing hypertension and cardiovascular disease (CVD) in subsequent years (7). Therefore, accurately identifying individuals in the early stages of hypertension and initiating timely drug treatment and lifestyle interventions are crucial for mitigating the risk of hypertension-related clinical complications and CVD, as well as reducing the likelihood of cardiovascular-related mortality.

The atherogenic index of plasma (AIP) is a well-established biomarker for assessing plasma atherosclerosis (8). Numerous existing studies have consistently demonstrated a close association between AIP and a range of diseases, including cardiovascular disease (CVD), insulin resistance, and diabetes (9, 10). Notably, research conducted by Qin Minghui et al. (11) revealed a significant positive correlation between AIP and both cardiovascular disease mortality (CVM) and all-cause mortality (ACM), with this relationship being particularly evident in middle-aged and older adults. Zheng Yitian (12) and his colleagues observed that individuals with a higher atherogenic index of plasma (AIP) had a significantly elevated likelihood of experiencing major adverse cardiac events (MACE) compared to those with a lower AIP index. Furthermore, there exists a distinct ‘J’-shaped relationship between AIP and the risk of MACE occurrence. Hypertension, being one of the more prevalent cardiovascular diseases, has seen relatively limited research examining its correlation with AIP, and even fewer studies have focused on the relationship between pre-hypertension and AIP. Early identification and detection within the pre-hypertensive population hold substantial clinical and societal value in preventing the progression of hypertension and more severe cardiovascular diseases.

Recent studies have demonstrated that abnormal AIP is closely linked to hormonal metabolism, with postmenopausal women exhibiting a higher susceptibility to elevated AIP levels (13, 14). The direct decline in estrogen and inactivation of estrogen receptor signaling pathways directly or indirectly suppress high-density lipoprotein cholesterol (HDL-C) synthesis and impair triglyceride (TG) hydrolysis. Furthermore, female monocyte-macrophages display heightened sensitivity to pro-inflammatory responses in high-AIP environments compared to males. Additionally, genome-wide analyses reveal that DNA methylation modifications at AIP-associated loci (e.g., APOA5, LPL) in females are significantly correlated with systolic blood pressure, whereas no such association is observed in males, Some studies have even suggested that the AIP may serve as a robust independent predictor of coronary artery disease (CAD) risk in Han Chinese postmenopausal women, potentially outperforming conventional lipid parameters (15, 16). These findings suggest that the impact of elevated AIP on blood pressure regulation may be more pronounced in the female population.

Therefore, our study implemented a dual-phase analytical framework integrating cross-sectional and longitudinal methodologies, leveraging nationally representative data from the China Health and Retirement Longitudinal Study (CHARLS), to systematically evaluate the dose-response relationship between atherogenic index of plasma (AIP) trajectories and the progression from normotension to pre-hypertension/hypertension in Chinese adults aged ≥45 years. Crucially, we incorporated sex-stratified mediation analyses to elucidate the mechanistic divergence in AIP-associated cardiovascular pathophysiology between genders.

## 2 Methods

### 2.1 Research design and included subjects

All the subjects we study are sourced from the CHARLS database. CHARLS is a prospective longitudinal study project led by the National School of Development at Pekingg University, primarily focusing on the Chinese population aged 45 and above and their spouses. The research covers not only their health status but also delves into the social and economic backgrounds of the residents (17). This study used a multi-stage probability sampling technique to screen the research subjects to ensure that the selected samples are representative. The study began with baseline data collection in 2011, covering 28 provinces in mainland China, with 17,708 participants. The main contents of the survey include biannual questionnaire follow-ups, physical measurements, and the collection and analysis of hematological samples conducted once every two follow-up periods (17, 18).

**Figure 1:**
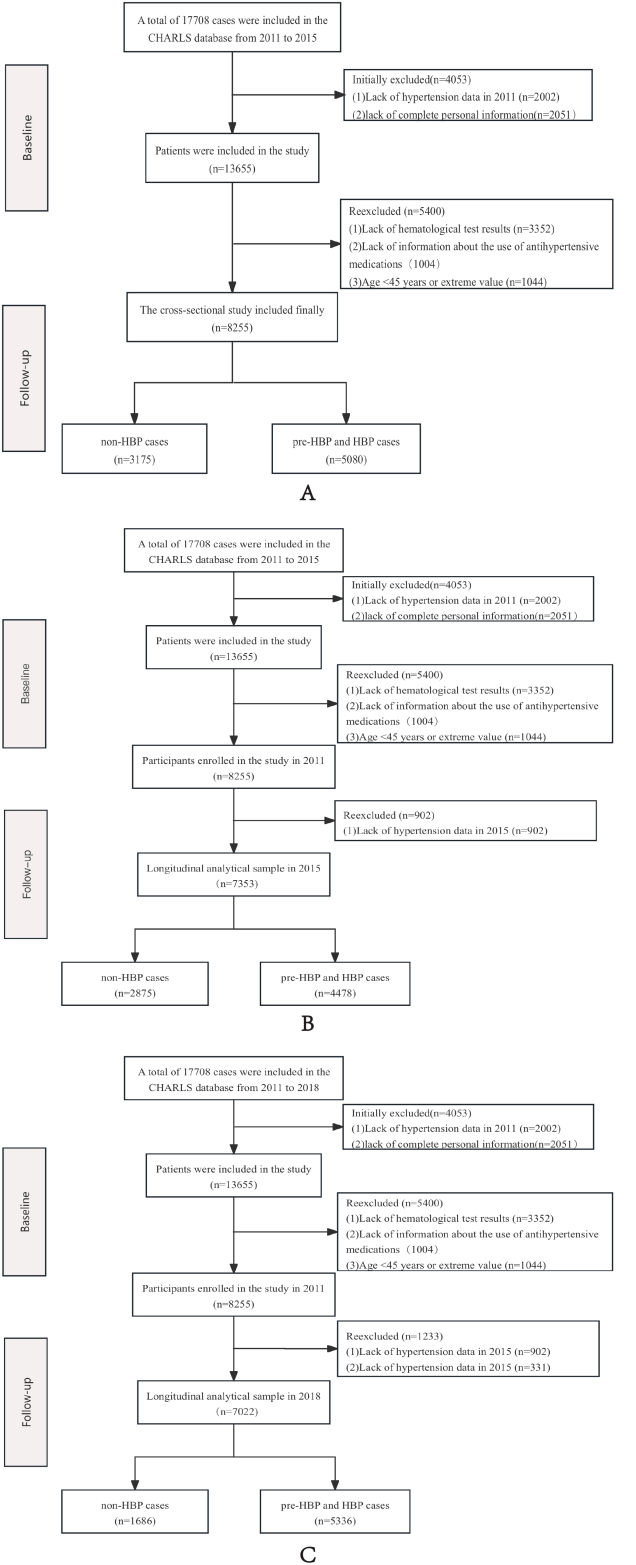
The flowchart of study participants. **(A)** Flow chart of the cross-sectional study, **(B)** Flow chart of the longitudinal study in 2015, **(C)** Flow chart of the longitudinal study in 2018.

In this cross-sectional study, we enrolled 17708 participants in 2011. And then, we excluded 2002 participants due to missing hypertension-related information, 4535 with incomplete demographic data, 3352 lacking hematological test results, and an additional 1004 with incomplete datasets. After further excluding 9453 individuals aged below 45 years, the final included population had an age range of 45 to 102 years. Subsequent comprehensive analysis focused on the remaining 8255 participants: 3175 normotensive individuals and 5080 with pre-hypertension or hypertension. Building on these findings, we conducted a longitudinal study after excluding 902 participants lacking 2015 hypertension follow-up data. The final cohort comprised 7353 subjects, including 2875 normotensive individuals and 4478 with pre-hypertension or hypertension, compared with baseline, the overall response rate was 87.15%. In the course of the longitudinal cohort study in 2018, a total of 7022 subjects were enrolled, including 1686 subjects with normal blood pressure and 7022 subjects with pre-hypertension or hypertension, and the overall response rate was 86.46% (**Figure 1**).

### 2.2 Definition of exposure factors and outcome variables

The exposure factor in the study is AIP, defined as AIP = log10 [TG (mg/dL)/HDL-C (mg/dL)] (10, 19), the recommended normal reference range for the AIP index is -0.3 to 0.1, a value between 0.1 and 0.24 is considered moderate risk, and a value greater than 0.24 is classified as high risk. The determination of pre-hypertension, which refers to elevated normal blood pressure, is based on the diagnostic criteria outlined in the “Chinese Guidelines for the Prevention and Treatment of Hypertension (2018 Revised Edition)” (20). The pre-hypertension blood pressure range is defined as a systolic pressure greater than 120 mmHg and less than or equal to 139 mmHg and/or a diastolic pressure greater than 80 mmHg and less than or equal to 89 mmHg; the definition of hypertension is a systolic blood pressure >140 mmHg and/or diastolic blood pressure >90 mmHg or currently taking anti-hypertensive medication (20, 21).

The blood pressure data was measured by the interviewer using a specialized automatic blood pressure monitor (OmronTM HEM-7200, manufactured by Omron (Dalian) Co., Ltd.) or a calibrated mercury sphygmomanometer from CHNS, with a total of three measurements taken at 45-second intervals. The final blood pressure value included in the study is the average of the three measurements (22).

### 2.3 Covariates

This study considered multiple covariates, including categorical variables (such as sex, smoking status, alcohol consumption, diabetes status, dyslipidemia status, and education level) and continuous variables, such as age, blood pressure, body mass index(BMI), triglycerides(TG), total cholesterol(TC), low-density lipoprotein(LDL), high-density lipoprotein(HDL), blood glucose(BG), and hemoglobinA1c (HbA1c), and the use of antihypertensive drugs and antilipemic drugs. All information provided by the respondents was measured and recorded by professionally trained interviewers using a standardized questionnaire. The hematological indicators were collected by staff from the Chinese Center for Disease Control and Prevention (China CDC) after the subjects had fasted for more than 8 hours, and measurements were taken according to the prescribed procedures (18, 22, 23). They use enzymatic colorimetry to measure blood glucose and lipids, while HbA1c is measured through boronate affinity high-performance liquid chromatography (24).

### 2.4 Statistical analysis

Statistical analysis of the data was conducted using version 27.0 of SPSS software (IBM SPSS, Armonk, NY, USA), and GraphPad Prism version 9.5.1 was used for plotting (GraphPad Prism 9.5.1 Macintosh Version by Software MacKiev ^©^ 1994-2023 GraphPad Software, LLC). All statistical tests are two-tailed, with a *P*-value less than 0.05 considered statistically significant. Continuous variables are expressed as median and mean ± standard deviation, while categorical variables are presented as frequency and percentage. Differences between groups are compared using the Kruskal-Wallis H test, one-way analysis of variance, and the chi-square test.

This study employed multivariate logistic regression models to analyze the association between AIP exposure and the development of pre-hypertension and hypertension, with risk quantified using adjusted odds ratios (ORs) and 95% confidence intervals (CIs). Model 1 utilized unadjusted data, while Model 2 was adjusted for covariates including sex, age, educational level, diabetes status, smoking and alcohol consumption habits, and BMI. Building on Model 2, Model 3 further incorporated adjustments for additional factors such as LDL-C, TC, BG, HbA1c, and the use of antihypertensive and lipid-lowering medications. A generalized additive model with restricted cubic splines (RCS) was constructed to evaluate the dose-response relationship between atherogenic index of plasma (AIP) exposure and the development of pre-hypertension and hypertension. Additionally, stratified analyses were performed to assess the modifying effects of sex and menopausal status on this association.

## 3 Results

The baseline study included 8255 participants, comprising 3740 males (45.31%) and 4515 females (54.69%), with a mean age of 60.17 ± 8.75 years. Among them, 2829 cases (34.3%) were classified as prehypertensive and 2251 cases (27.2%) as hypertensive. Demographic and clinical characteristics of the study population were compared through stratified analysis based on baseline AIP quartiles (**Table 1**). Notably, all variables, except for daily cigarette consumption, exhibited statistical significance across the four AIP categories. Participants with higher AIP levels generally demonstrated higher BMI values, elevated blood pressure (both systolic and diastolic), and increased BG levels compared to those with lower AIP levels.

**Table 1:** Demographic and clinical characteristics of the study population.

Variable	AIP Quartiles					*P* value
	Total	Q1 (< 0.11)	Q2 (0.11 to 0.22)	Q3 (0.22 to 0.31)	Q4 (≥ 0.31)	
Participants(n)	8255	2063	2065	2063	2064	
Sex						
Female(n, %)	4633(53.1%)	1028(49.8%)	1142(55.3%)	1161(56.3%)	1184(57.4%)	<0.001
Male(n, %)	3850(46.9%)	1035(50.2%)	923(44.7%)	902(43.7%)	880(42.6%)	
Age(year)	60.17±8.75	63.02±9.56	62.41±9.67	61.39±9.17	59.78±8.79	<0.001
BMI(kg/m^2^)	24.35±6.94	22.81±8.74	23.77±8.6	25.37±6.88	25.48±6.91	<0.001
<18.5	538(6.5%)	238(11.5%)	164(7.9%)	88(4.3%)	48(2.3%)	<0.001
18.5-23.9	3121(37.8%)	1032(50%)	852(41.3%)	723(35%)	514(24.9%)	
24-24.9	1715(20.8%)	380(18.4%)	438(21.1%)	429(20.8%)	468(22.7%)	
≥25	2766(33.5%)	388(18.8%)	584(28.3%)	791(38.3%)	1003(48.6%)	
SBP(mmHg)	128.2±20.43	121.57±19.40	127.43±17.89	130.80±18.78	133.04±18.02	<0.001
DBP(mmHg)	88.19±9.07	86.32±7.93	87.98±7.43	89.04±8.71	89.95±9.11	<0.001
HBP state						
none-hypertension	3175(38.5%)	901(43.7%)	859(41.6%)	764(37%)	651(31.5%)	<0.001
pre-hypertension	2829(34.3%)	670(32.5%)	697(33.8%)	705(34.2%)	757(36.7%)	
hypertension	2251(27.2%)	492(23.8%)	509(24.6%)	594(28.8%)	656(31.8%)	
HBP Classification						
Grade 1	1641(72.9%)	374(76%)	382(75%)	426(71.7%)	459(70%)	<0.001
Grade 2	394(17.5%)	83(16.9%)	82(16.1%)	106(17.8%)	123(18.8%)	
Grade 3	216(9.6%)	35(7.1%)	45(8.9%)	62(10.5%)	74(11.2%)	
TC(mg/dL)	184.39±35.66	181.15±34.07	185.37±34.43	189.47±35.30	196.88±41.57	<0.001
TG(mg/dL)	138.49±19.97	74.15±17042	108.83±20.59	156.98±31.14	296.61±22.71	<0.001
HDL-C(mg/dL)	51.73±11.76	61.41±12.14	52.05±8.05	47.56±8.14	42.55±7.58	<0.001
LDL-C(mg/dL)	103.14±28.54	101.20±28.21	109.72±28.43	109.57±28.43	112.37±29.17	<0.001
Blood glucose(mg/dL)	103.29±34.79	96.03±25.97	99.87±24.44	104.19±27.97	117.35±28.13	<0.001
HbA1c(%)	5.99±0.98	5.79±0.67	5.94±0.83	6.01±0.97	6.27±1.32	<0.001
Current smoking(n, %)	1895(22.96%)	516(25.0%)	454(22.0%)	486(23.7%)	439(%)	0.002
Cigarettes intake per day						
<5	151(7.97%)	41(7.95%)	37(8.15%)	34(7.0%)	39(8.9%)	0.812
5 to 20	968(51.08%)	272(52.71%)	223(49.12%)	277(57.0%)	196(44.6%)	
≥20	776(40.95%)	203(39.34%)	194(42.73%)	175(36.0%)	204(46.5%)	
Current drinking(n, %)	2356(28.54%)	657(35.25%)	654(34.86%)	524(27.87%)	521(25.24%)	<0.001
Alcohol intake per day						
<2	1867(82.4%)	500(76.1%)	485(74.16%)	437(83.4%)	445(85.4%)	<0.001
≥2	399(17.6%)	157(23.9%)	79(25.84%)	87(16.6%)	76(14.59%)	
Diabetes						
Yes	1034(12.5%)	134(6.5%)	208(10.1%)	294(14.3%)	398(19.3%)	<0.001
No	6471(78.4%)	1732(84.0%)	1671(80.9%)	1589(77.0%)	1479(71.7%)	
Dyslipidemia						
Yes	1768(21.4%)	262(12.7%)	340(16.5%)	511(24.8%)	655(31.7%)	<0.001
No	5736(69.5%)	1604(77.8%)	1539(74.5%)	1371(66.5%)	1222(59.2%)	
Take medicine for HBP						
Yes	2360 (28.6%)	434 (21.0%)	486 (23.5%)	675 (32.7%)	765 (37.1%)	<0.001
No	4992 (60.5%)	1394 (67.6%)	1350 (65.4%)	1171 (56.8%)	1077 (52.2%)	
Take medicine for hyperlipidemia						
Yes	947 (11.5%)	122 (5.9%)	199 (9.6%)	263 (12.8%)	363 (17.6%)	<0.001
No	6557 (79.4%)	1744 (84.5%)	1680 (81.4%)	1619 (78.5%)	1514 (73.4%)	

Regardless of covariate adjustments, AIP is significantly positively associated with the incidence of pre-hypertension and hypertension among study participants. We divided AIP into 4 groups according to quartiles, using Q1 as the baseline group to evaluate the association between AIP and pre-hypertension and hypertensive disorders. Covariates such as sex, age, education level, history of smoking and drinking, diabetes, BMI, TC, LDL-C, HbA1c, and use of anti-hypertensive and lipid-lowering drugs were adjusted.

**Figure 2:**
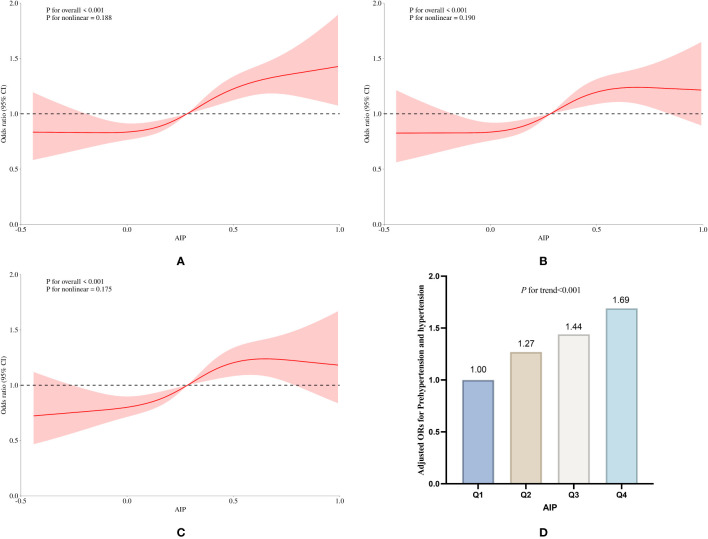
Association between AIP and prevalence of pre-hypertension and hypertension in the cross-sectional study. **(A)** RCS curves without adjustment for covariates, **(B)** RCS curves after adjusting for covariates such as sex, education, age, smoking and drinking status, and BM, **(C)** RCS curves after adjusting for covariates such as TC, LDL-C, BG, and HbA1c, the use of antihypertensive drugs and blood-lipid lowering drugs based on **(B)**; **(D)** Relative odds of pre-hypertension and hypertension corresponding to quartiles of AIP.

In the cross-sectional study, compared with the reference group of Q1, the ORs for the development of pre-hypertension and hypertension in the other groups were Q2 (OR: 1.27, 95%CI: 1.06 to 1.51, *P*=0.008), Q3 (OR: 1.44, 95%CI: 1.20 to 1.74, *P*<0.001), and Q4 (OR: 1.69, 95%CI: 1.38 to 2.07, *P*<0.001), *P* for trend was less than 0.001 (**Table 2**, **Figure 2**).

**Table 2:** The relative risks of pre-hypertension and hypertension in all subjects were calculated according to different AIP models in the cross-sectional study of 2011.

AIP	Pre-hypertension and hypertension OR (95%CI)					
	Model 1		Model 2		Model 3	
	OR (95%CI)	*P* value	OR (95%CI)	*P* value	OR (95%CI)	*P* value
Per-SD increase	1.81(1.62,2.07)	<0.001	1.74(1.55,1.91)	<0.001	1.51(1.34,1.77)	<0.001
Quartiles						
Q1(< 0.11)	1.00(Reference)		1.00(Reference)		1.00(Reference)	
Q2(0.11 to 0.22)	1.18 (1.02,1.37)	0.026	1.23 (1.05,1.43)	0.01	1.27 (1.06,1.51)	0.008
Q3(0.22 to 0.31)	1.38 (1.19,1.60)	<0.001	1.42 (1.21,1.66)	<0.001	1.44 (1.20,1.74)	<0.001
Q4(≥ 0.31)	1.59 (1.36,1.85)	<0.001	1.64 (1.39,1.94)	<0.001	1.69 (1.38,2.07)	<0.001
*P* for trend		<0.001		<0.001		<0.001

OR, Odds Ratio; CI, Confidence Interval.

Model 1: was adjusted for none.

Model 2: was adjusted for age, sex, education, diabetes, current smoking, alcohol intake and BMI.

Model 3: was adjusted for age, sex, education, diabetes, current smoking, alcohol intake, BMI, LDL_C, TC, blood glucose, HbA1c, the use of antihypertensive drugs and blood-lipid lowering drugs.

**Figure 3:**
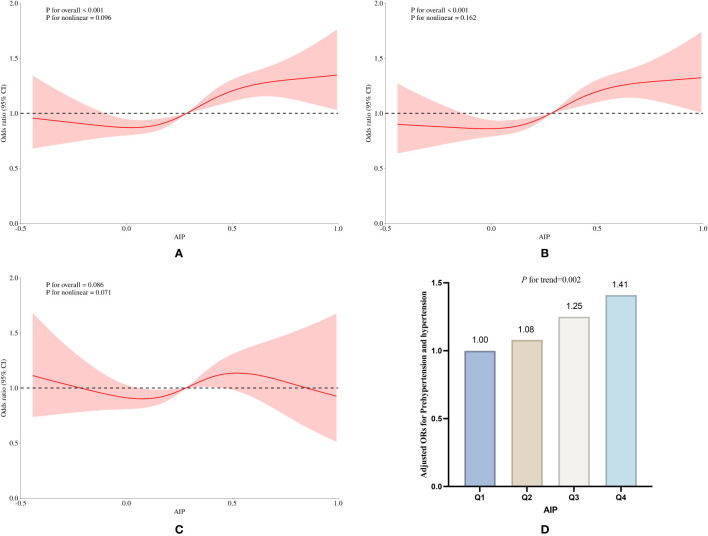
Association between AIP and prevalence of pre-hypertension and hypertension in the longitudinal studies of 2018. **(A)** RCS curves without adjustment for covariates, **(B)** RCS curves after adjusting for covariates such as age, sex, education, age, smoking and drinking status, and BMI and income, **(C)** RCS curves after adjusting for covariates such as LDL_C, TC, such as TC, LDL-C, BG, and HbA1c, the use of antihypertensive drugs and blood-lipid lowering drugs based on **(B)**; **(D)** Relative odds of pre-hypertension and hypertension corresponding to quartiles of AIP.

In the longitudinal study of 2015, we found that compared with Q1, the odds ratios for pre-hypertension and hypertension in the other groups were Q2 (OR:1.10, 95%CI: 0.93 to 1.30, *P*=0.257), Q3 (OR:1.20, 95%CI: 1.01 to 1.43, *P*=0.045) and Q4 (OR:1.38, 95%CI: 1.13 to 1.67, *P*=0.001). This trend is also completely consistent with the results of the RCS curve(**Supplementary Figure S1**, **Supplementary Table S1**). In the 2018 longitudinal cohort, a dose-dependent elevation in pre-hypertension and hypertension risk was observed with increasing index of AIP levels. Compared to baseline, Q2 exhibited a 1.08-fold risk increase (95% CI:0.93 to 1.26, P=0.296), Q3 a 1.25-fold increase (95% CI: 1.07 to 1.46, *P*=0.005), and Q4 demonstrated the most pronounced risk escalation (OR:1.41,95% CI:1.20 to 1.65, *P*<0.001). In summary, our findings demonstrate a dose-dependent increase in the incidence of pre-hypertension and hypertension with rising AIP levels, revealing a statistically significant correlation between these parameters (*P* for trend <0.001) (**Figure 3**, **Table 3**).

**Table 3:** The relative risks of pre-hypertension and hypertension in all subjects were calculated according to different AIP models in 2018.

AIP	Pre-hypertension and hypertension OR (95%CI)					
	Model 1		Model 2		Model 3	
	OR (95%CI)	*P* value	OR (95%CI)	P value	OR (95%CI)	*P* value
Per-SD increase	1.61(1.30,1.77)	<0.001	1.50(1.38,1.71)	0.019	1.31(1.18,1.47)	0.034
Quartiles						
Q1(< 0.11)	1.00(Reference)		1.00(Reference)		1.00(Reference)	
Q2(0.11 to 0.22)	1.05 (0.92,1.20)	0.41	1.10 (0.96,1.27)	0.182	1.08 (0.93,1.26)	0.296
Q3(0.22 to 0.31)	1.21 (1.05,1.39)	0.018	1.27 (1.10,.47)	0.001	1.25 (1.07,1.46)	0.005
Q4(≥0.31)	1.47 (1.28,1.70)	<0.001	1.61 (1.38,1.87)	<0.001	1.41 (1.20,1.65)	<0.001
*P* for trend		0.013		0.004		0.002

OR, Odds Ratio; CI, Confidence Interval.

Model 1: was adjusted for none.

Model 2: was adjusted for education, diabetes, current smoking, alcohol intake and BMI.

Model 3: was adjusted for education, diabetes, current smoking, alcohol intake, BMI, LDL_C, TC, blood glucose, HbA1c, the use of antihypertensive drugs and blood-lipid lowering drugs.

**Figure 4:**
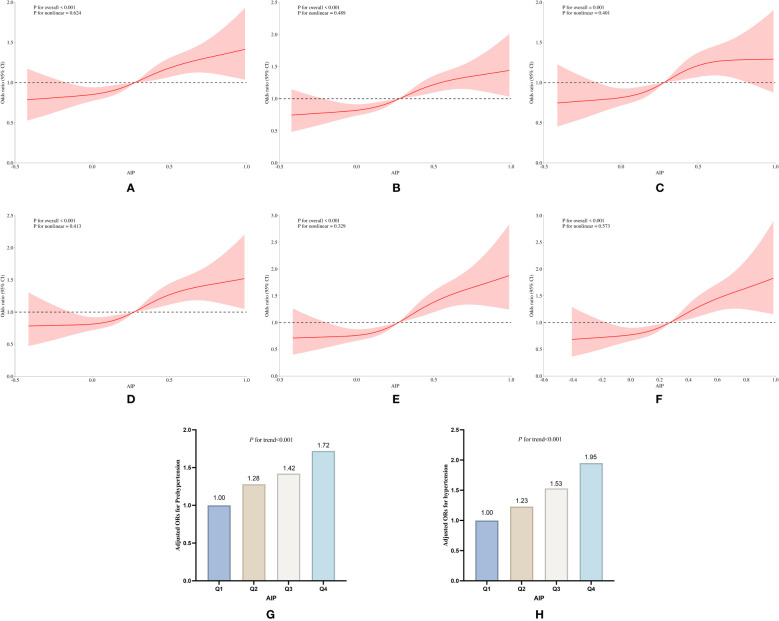
Subgroup analysis of the association of AIP with pre-hypertension and hypertension prevalence in the cross-sectional study. **(A)** RCS curves of AIP and pre-hypertension incidence without adjustment for covariates, **(B)** RCS curves of AIP and pre-hypertension incidence after adjusting for covariates such as age, sex, education, age, smoking and drinking status, and BMI, **(C)** RCS curves of AIP and pre-hypertension incidence after adjusting for covariates such as LDL_C, TC, blood glucose, HbA1c, the use of anti-hypertensive drugs and blood-lipid lowering drugs based on **(B)**; **(D)** RCS curves of AIP and hypertension incidence without adjustment for covariates; **(E)** RCS curves of AIP and hypertension incidence after adjusting for covariates such as age, sex, education, age, smoking and drinking status, and BMI; **(F)** CS curves of AIP and pre-hypertension incidence after adjusting for covariates such as LDL_C, TC, blood glucose, HbA1c, the use of antihypertensive drugs and blood-lipid lowering drugs based on **(E)**; **(G, H)** Relative odds of pre-hypertension and hypertension corresponding to quartiles of AIP.

Both cross-sectional and longitudinal studies have demonstrated that exposure to high levels of AIP increases the incidence of pre-hypertension and hypertension. In the cross-sectional study, individuals in the highest AIP exposure group exhibited a 72% increase in the incidence of pre-hypertension (OR: 1.72, 95% CI: 1.37 to 2.17, *P*<0.001) compared to those in the lowest exposure group. Additionally, the risk of developing hypertension increased by 95% (OR: 1.95, 95% CI: 1.44 to 2.64, *P*<0.001) in the highest exposure group. In the cross-sectional study, after adjusting for the covariates including sex, age, education level, smoking history, drinking history, diabetes, BMI, TC, LDL-C, HbA1c, as well as antihypertensive and lipid-lowering medications, we observed that the risk of developing hypertension in the Q2, Q3, and Q4 groups was significantly increased compared to the Q1 group. Specifically, the ORs and 95% CIs for the Q2, Q3, and Q4 groups were 1.04 (95% CI: 0.85 to 1.29), 1.28 (95% CI: 1.03 to 1.58), and 1.62 (95% CI: 1.30 to 2.03), respectively. Notably, except for the Q2 group, statistically significant differences were found between AIP exposure and the incidence of hypertension in both the Q3 and Q4 groups (**Table 4**, **Figure 4**).

**Table 4:** Multivariate logistic regression analysis of the relationship between AIP and pre-hypertension as well as hypertension in cross-sectional study.

AIP	Model 1		Model 2		Model 3	
	OR (95%CI)	*P* value	OR (95%CI)	*P* value	OR (95%CI)	*P* value
Pre-hypertension						
Per-SD increase	1.54(1.37,1.77)	<0.001	1.68(1.45,1.92)	<0.001	1.71 (1.55,2.07)	<0.001
Quartiles						
Q1(< 0.11)	1.00(Reference)		1.00(Reference)		1.00(Reference)	
Q2(0.11 to 0.22)	1.18 (1.01,1.39)	0.048	1.23 (1.04,1.47)	0.018	1.28 (1.05,1.56)	0.014
Q3(0.22 to 0.31)	1.31 (1.10,1.54)	0.002	1.38 (1.15, 1.65)	<0.001	1.42 (1.15,1.76)	0.001
Q4(≥ 0.31)	1.53 (1.29,1.81)	<0.001	1.66 (1.38,2.00)	<0.001	1.72 (1.37,2.17)	<0.001
*P* for trend		<0.001		<0.001		<0.001
Hypertension						
Per-SD increase	1.65(1.33,2.18)	<0.001	1.68(1.54,2.22)	0.001	1.81 (1.66,2.21)	0.037
Quartiles						
Q1(< 0.11)	1.00(Reference)		1.00(Reference)		1.00(Reference)	
Q2(0.11 to 0.22)	1.18 (0.96,1.46)	0.125	1.25 (0.99,1.59)	0.066	1.23 (0.94,1.61)	0.127
Q3(0.22 to 0.31)	1.47 (1.19,1.81)	<0.001	1.65 (1.30,2.09)	<0.001	1.53 (1.15,2.03)	0.004
Q4(≥ 0.31)	1.67 (1.34,2.07)	<0.001	2.08 (1.63,2.66)	<0.001	1.95 (1.44, 2.64)	<0.001
*P* for trend		<0.001		<0.001		<0.001
*P* value for interaction		<0.001		<0.001		<0.001

OR, Odds Ratio; CI, Confidence Interval.

Model 1: was adjusted for none.

Model 2: was adjusted for age, education, diabetes, current smoking, alcohol intake and BMI.

Model 3: was adjusted for age, education, diabetes, current smoking, alcohol intake, BMI, LDL_C, TC, blood glucose, HbA1c, the use of antihypertensive drugs and blood-lipid lowering drugs.

**Figure 5:**
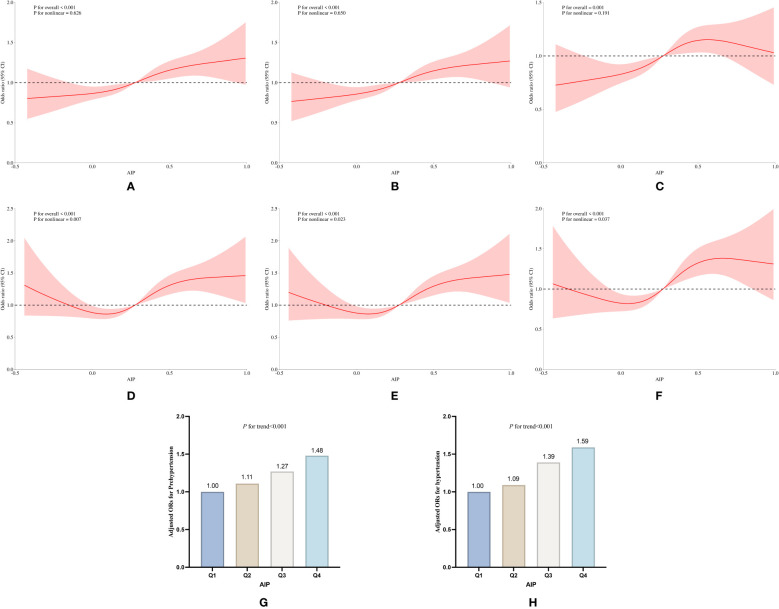
Subgroup analysis of the association of AIP with pre-hypertension and hypertension prevalence in the Longitudinal study of 2018. **(A)** RCS curves of AIP and pre-hypertension incidence without adjustment for covariates, **(B)** RCS curves of AIP and pre-hypertension incidence after adjusting for covariates such as age, sex, education, age, smoking and drinking status, and BMI, **(C)** RCS curves of AIP and pre-hypertension incidence after adjusting for covariates such as LDL_C, TC, blood glucose, HbA1c, the use of anti-hypertensive drugs and blood-lipid lowering drugs based on **(B)**; **(D)** RCS curves of AIP and hypertension incidence without adjustment for covariates; **(E)** RCS curves of AIP and hypertension incidence after adjusting for covariates such as age, sex, education, age, smoking and drinking status, and BMI; **(F)** CS curves of AIP and pre-hypertension incidence after adjusting for covariates such as LDL_C, TC, blood glucose, HbA1c, the use of antihypertensive drugs and blood-lipid lowering drugs based on **(E)**; **(G, H)** Relative odds of pre-hypertension and hypertension corresponding to quartiles of AIP.

In longitudinal cohort studies, individuals with the highest exposure to the AIP index exhibited a 51% increased risk of developing pre-hypertension in 2015 (OR:1.51, 95% CI: 1.24 to 1.83, *P*<0.001) and a 62% elevated risk of hypertension (OR:1.62, 95% CI: 1.30 to 2.03, *P*<0.001) (**Supplementary Table S2**, **Supplementary Figure S2**). In the 2018 longitudinal cohort, these risks were 48% (OR: 1.48, 95% CI: 1.23 to 1.77, *P*<0.001) for pre-hypertension and 59% (OR:1.59, 95% CI: 1.30 to 1.99, *P*<0.001) for hypertension (**Table 5**, **Figure 5**). As demonstrated across both the 2011 cross-sectional study and longitudinal cohort studies in 2015 and 2018, elevated atherogenic index of plasma levels were consistently associated with a progressive rise in the risks of hypertension and pre-hypertension (*P* for trend <0.05).

**Table 5:** Multivariate logistic regression analysis of the relationship between AIP and pre-hypertension as well as hypertension in 2018.

AIP	Model 1		Model 2		Model 3	
	OR (95%CI)	*P* value	OR (95%CI)	*P* value	OR (95%CI)	*P* value
Pre-hypertension						
Per-SD increase	1.41(1.22,1.87)	0.016	1.50(1.33,1.82)	0.007	1.61(1.37,1.90)	0.002
Quartiles						
Q1(< 0.11)	1.00(Reference)		1.00(Reference)		1.00(Reference)	
Q2(0.11 to 0.22)	1.18 (1.03,1.45)	0.047	1.10 (1.01,1.30)	0.252	1.11 (0.94,1.32)	0.233
Q3 (0.22 to 0.30)	1.28 (1.121,1.59)	0.013	1.21 (1.12,1.40)	0.03	1.27 (1.16,1.52)	0.016
Q4 (≥0.31)	1.55 (1.29, 1.81)	<0.001	1.45 (1.22,1.71)	<0.001	1.48 (1.23,1.77)	<0.001
*P* for trend		0.035		0.019		<0.001
Hypertension						
Per-SD increase	1.43(1.24,1.66)	0.023	1.51(1.24,1.79)	0.018	1.61(1.41,2.01)	0.002
Quartiles						
Q1(< 0.11)	1.00(Reference)		1.00(Reference)		1.00(Reference)	
Q2(0.11 to 0.22)	1.09 (1.03,1.29)	0.27	1.03 (0.85,1.25)	0.756	1.09 (0.88,1.36)	0.441
Q3(0.22 to 0.31)	1.44 (1.27,1.76)	0.015	1.25 (1.03,1.53)	0.025	1.39 (1.12,1.74)	0.003
Q4(≥0.31)	1.51 (1.35,1.88)	<0.001	1.55 (1.27,1.89)	<0.001	1.59 (1.27,1.99)	<0.001
*P* for trend		0.002		0.001		<0.001
*P* value for interaction		<0.001		<0.001		<0.001

OR, Odds Ratio; CI, Confidence Interval.

Model1: was adjusted for none.

Model2: was adjusted for age, education, diabetes, current smoking, alcohol intake and BMI.

Model3: was adjusted for age, education, diabetes, current smoking, alcohol intake, BMI, LDL_C, TC, blood glucose, HbA1c, the use of antihypertensive drugs and blood-lipid lowering drugs.

**Figure 6:**
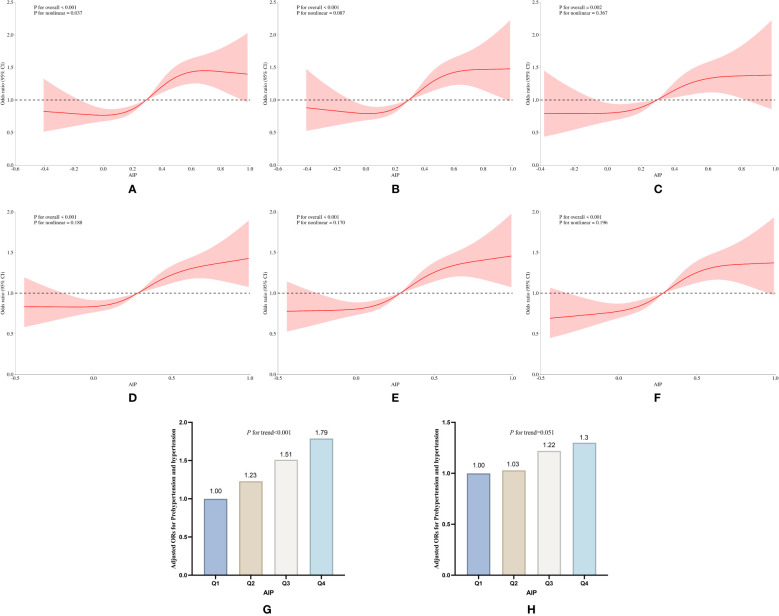
Subgroup analysis of the association of AIP with pre-hypertension and hypertension prevalence between sexes in the cross-sectional study. **(A)** RCS curves of AIP versus pre-hypertension and hypertension in females without covariate adjustment, **(B)** RCS curves of AIP and prevalence of pre-hypertension and hypertension in females after adjusting for age, education, age, smoking, and drinking status, and BMI, **(C)** RCS curves of AIP and prevalence of pre-hypertension and hypertension in females after adjusting for covariates such as LDL_C, TC, blood glucose, HbA1c, using of antihypertensive drugs and blood-lipid lowering drugs based on **(B)**; **(D)** RCS curves of AIP versus pre-hypertension and hypertension in males without covariate adjustment; **(E)** RCS curves of AIP and prevalence of pre-hypertension and hypertension in males after adjusting for age, education, age, smoking, and drinking status, and BMI, **(F)** RCS curves of AIP and prevalence of pre-hypertension and hypertension in males after adjusting for covariates such as LDL_C, TC, blood glucose, HbA1c, the use of antihypertensive drugs and blood-lipid lowering drugs based **(E)**; **(G, H)** Relative odds of pre-hypertension and hypertension in male and female groups corresponding to quartiles of AIP.

Our study revealed significant sex-specific differences in the relationship between AIP exposure and the development of pre-hypertension and hypertension. To investigate this, we validated the two independent components used to calculate AIP, HDL-C and TG, and found that only AIP itself exhibited sex-specific variations. Detailed subgroup analyses demonstrated that as AIP levels increased across different models, the risk of these conditions became markedly more pronounced in women, with an OR of 1.79 (95% CI:1.35 to 2.38, *P=*0.001). In the fully adjusted Model 3, elevated AIP levels in men showed no statistically significant association with pre-hypertension or hypertension risk (*P*=0.051) (**Table 6**). The findings from the logistic multivariate regression analysis align well with those derived from the RCS curve, suggesting that AIP has a more notable impact on the risk of developing pre-hypertension and hypertension among females (**Figure 6**).

**Table 6:** Relative risk of pre-hypertension and hypertension in males and females under different AIP models in the cross-sectional study.

AIP	Pre-hypertension and hypertension OR (95%CI)					
	Model 1		Model 2		Model 3	
	OR (95%CI)	*P* value	OR (95%CI)	*P* value	OR (95%CI)	*P* value
Female						
Per-SD increase	2.09(1.63,2.51)	<0.001	1.89(1.71,2.14)	<0.001	1.69(1.33,2.07)	<0.001
Quartiles						
Q1(< 0.11)	1.00(Reference)		1.00(Reference)		1.00(Reference)	
Q2(0.11 to 0.22)	1.18 (1.02,1.37)	0.026	1.24 (1.06,1.46)	0.006	1.23 (1.03,1.47)	0.024
Q3(0.22 to 0.31)	1.55 (1.26,1.90)	<0.001	1.51 (1.21,1.90)	<0.001	1.51 (1.16,1.96)	<0.001
Q4(≥ 0.31)	1.83 (1.49,2.25)	<0.001	1.85 (1.48,2.33)	<0.001	1.79 (1.35,2.38)	<0.001
*P* for trend		<0.001		<0.001		<0.001
Male						
Per-SD increase	1.55(1.27,1.99)	<0.001	1.27(1.11,1.41)	0.003	1.11(1.01,1.40)	0.051
Quartiles						
Q1(< 0.11)	1.00(Reference)		1.00(Reference)		1.00(Reference)	
Q2(0.11 to 0.22)	1.08 (1.02, 1.27)	0.202	1.14 (1.06,1.36)	0.371	1.03 (1.03,1.47)	0.254
Q3(0.22 to 0.31)	1.18 (1.09, 1.40)	<0.001	1.25 (1.13,1.71)	<0.001	1.22 (1.08,1.653)	0.055
Q4(≥ 0.31)	1.39 (1.16, 1.55)	<0.001	1.43 (1.16,1.85)	<0.001	1.30 (1.17,1.79)	0.006
*P* for trend		<0.001		<0.001		0.051
*P* value for interaction		<0.001		<0.001		0.001

OR, Odds Ratio; CI, Confidence Interval.

Model1: was adjusted for none.

Model2: was adjusted for age, education, diabetes, current smoking, alcohol intake and BMI.

Model3: was adjusted for age, education, diabetes, current smoking, alcohol intake, BMI, LDL_C, TC, blood glucose, HbA1c, the use of antihypertensive drugs, and blood-lipid lowering drugs.

**Figure 7:**
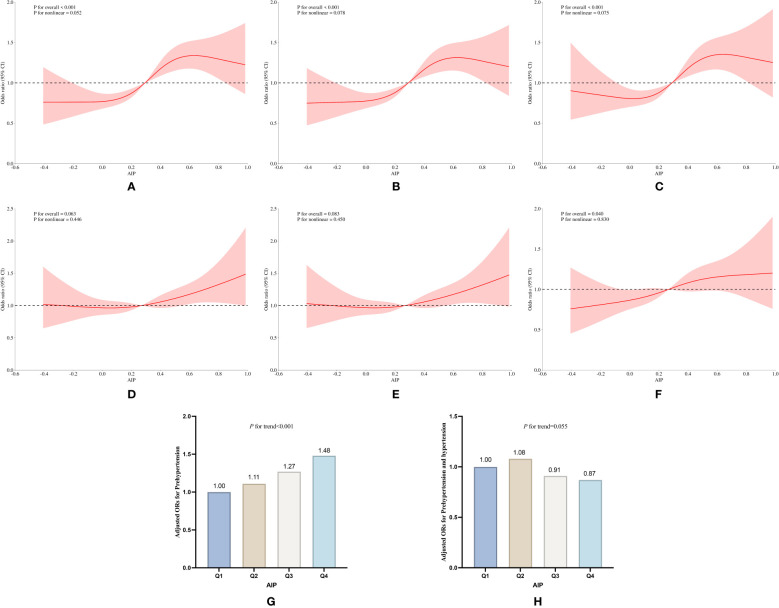
Subgroup analysis of the association of AIP with pre-hypertension and hypertension prevalence between the sexes in the longitudinal study of 2018. **(A)** RCS curves of AIP versus pre-hypertension and hypertension in females without covariate adjustment, **(B)** RCS curves of AIP and prevalence of pre-hypertension and hypertension in females after adjusting for age, education, age, smoking, and drinking status, and BMI, **(C)** RCS curves of AIP and prevalence of pre-hypertension and hypertension in females after adjusting for covariates such as LDL_C, TC, blood glucose, HbA1c, using of antihypertensive drugs and blood-lipid lowering drugs based on **(B)**; **(D)** RCS curves of AIP versus pre-hypertension and hypertension in males without covariate adjustment; **(E)** RCS curves of AIP and prevalence of pre-hypertension and hypertension in males after adjusting for age, education, age, smoking, and drinking status, and BMI, **(F)** RCS curves of AIP and prevalence of pre-hypertension and hypertension in males after adjusting for covariates such as LDL_C, TC, blood glucose, HbA1c, the use of antihypertensive drugs and blood-lipid lowering drugs based **(E)**; **(G, H)** Relative odds of pre-hypertension and hypertension in male and female groups corresponding to quartiles of AIP.

Stratified analyses conducted in 2015 and 2018 revealed a notable sex-specific pattern: females with elevated AIP exhibited a significantly higher risk of developing pre-hypertension and hypertension compared to their male counterparts. In the 2015 longitudinal cohort study, Model 3 revealed that females with the highest AIP exhibited a 49% increased risk of developing the aforementioned conditions compared to baseline levels (OR:1.49, 95% CI:1.14 to 1.95, *P*=0.003), while males showed only a 26% increase (OR:1.26, 95% CI:1.12 to 1.66, *P*=0.047) (**Supplementary Table S3**, **Supplementary Figure S3**). By 2018, this risk escalated to 64% in females (OR:1.64, 95% CI:1.31 to 2.06, *P*<0.001), whereas males even demonstrated a 13% reduction in risk (OR:0.87, 95% CI:0.57 to 1.31, *P*>0.05) (**Table 7**, **Figure 7**). Notably, the RCS curves and multivariable logistic regression analyses demonstrated findings essentially consistent with the aforementioned trends, further indicating that elevated AIP exerts a more pronounced impact on the risks of progressing to pre-hypertension and hypertension in female populations.

**Table 7:** Relative risk of pre-hypertension and hypertension in male and female under different AIP models in 2018.

AIP	Pre-hypertension and hypertension OR (95%CI)					
	Model 1		Model 2		Model 3	
	OR (95%CI)	*P* value	OR (95%CI)	*P* value	OR (95%CI)	*P* value
Female						
Per-SD increase	1.52(1.23,1.79)	<0.001	1.42(1.22,1.98)	<0.001	1.33(1.25,1.71)	0.004
Quartiles						
Q1(< 0.11)	1.00(Reference)		1.00(Reference)		1.00(Reference)	
Q2(0.11 to 0.22)	1.15 (1.04,1.42)	0.202	1.14 (0.94,1.38)	0.177	1.05 (0.85,1.30)	0.644
Q3(0.22 to 0.31)	1.41 (1.16,1.72)	<0.001	1.39 (1.14,1.69)	0.001	1.37 (1.10,1.71)	0.006
Q4(≥0.31)	1.73 (1.42,2.12)	<0.001	1.70 (1.39,2.09)	<0.001	1.64 (1.31,2.06)	<0.001
*P* for trend		<0.001		<0.001		<0.001
Male						
Per-SD increase	1.30(1.11,1.52)	0.003	1.17(1.06,1.51)	0.037	1.01(0.87,1.23)	0.145
Quartiles						
Q1(< 0.11)	1.00(Reference)		1.00(Reference)		1.00(Reference)	
Q2(0.11 to 0.22)	1.03 (0.84,1.25)	0.799	1.01 (0.83,1.24)	0.904	1.08 (0.77,1.23)	0.831
Q3(0.22 to 0.31)	1.06 (0.87,1.30)	0.559	1.06 (0.86,1.30)	0.611	0.91 (0.69,1.19)	0.492
Q4(≥ 0.31)	1.29 (1.04,1.60)	0.019	1.27 (1.02,.58)	0.033	0.87 (0.57,1.31)	0.497
*P* for trend		0.039		0.041		0.055
*P* value for interaction		0.002		0.023		0.047

OR, Odds Ratio; CI, Confidence Interval.

Model 1: was adjusted for none.

Model 2: was adjusted for age, education, diabetes, current smoking, alcohol intake and BMI.

Model 3: was adjusted for age, education, diabetes, current smoking, alcohol intake, BMI, LDL_C, TC, blood glucose, HbA1c, the use of antihypertensive drugs and blood-lipid lowering drugs.

**Figure 8:**
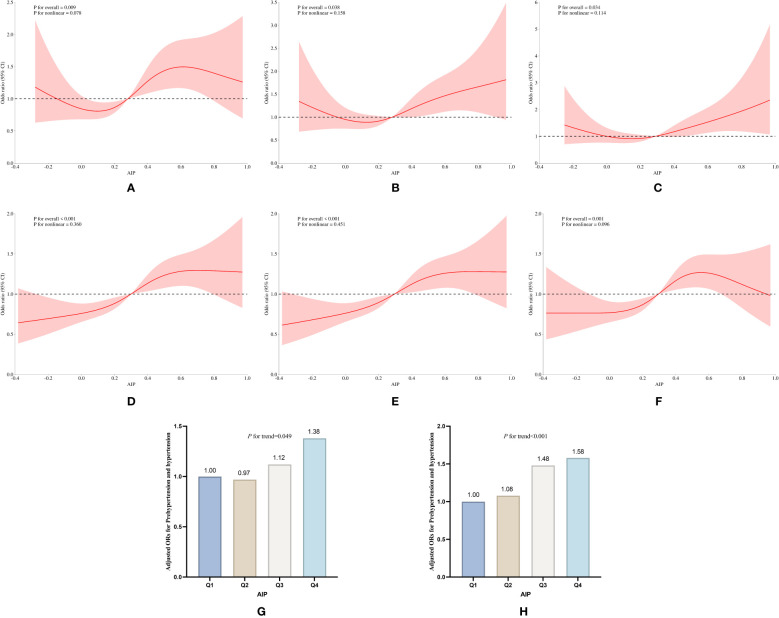
AIP and hypertension prevalence by menopausal status: 2018 subgroup analysis. **(A)** RCS curves of unadjusted AIP and pre-hypertension/hypertension prevalence in premenopausal women, **(B)** RCS curves of AIP and pre-hypertension/hypertension incidence after adjusting for covariates such as age, education, age, smoking and drinking status, and BMI in premenopausal women, **(C)** RCS curves of AIP and pre-hypertension/hypertension incidence after adjusting for covariates such as LDL_C, TC, blood glucose, HbA1c, the use of antihypertensive drugs and blood-lipid lowering drugs based on **(B)**; **(D)** RCS curves of unadjusted AIP and pre-hypertension/hypertension prevalence in postmenopausal women; **(E)** RCS curves of AIP and pre-hypertension/hypertension incidence after adjusting for covariates such as age,education, age, smoking and drinking status, and BMI in postmenopausal women, **(F)** RCS curves of AIP and pre-hypertension/hypertension incidence after adjusting for covariates such as LDL_C, TC, blood glucose, HbA1c, the use of antihypertensive drugs and blood-lipid lowering drugs based on **(E)**; **(G, H)** Relative odds of pre-hypertension/ hypertension corresponding to quartiles of AIP in premenopausal and postmenopausal women.

Considering the potential influence of menopausal status and hormone levels on AIP, we performed a stratified analysis by menopause status in the female population of the 2018 longitudinal cohort study. The results demonstrated that elevated AIP levels were associated with a higher risk of pre-hypertension and hypertension in postmenopausal women compared to premenopausal women. Among postmenopausal women, the highest AIP quartile (Q4) showed a 58% increased risk of developing pre-hypertension and hypertension relative to baseline (OR:1.58, 95% CI:1.20 to 2.07, *P*=0.001), whereas the corresponding risk increase in premenopausal women was 38% (OR:1.38, 95% CI:1.07 to 1.72, *P*=0.008) (**Table 8**, **Figure 8**).

**Table 8:** Relative risk of pre-hypertension and hypertension in postmenopausal and premenopausal female under different AIP models in 2018.

AIP	Pre-hypertension and hypertension OR (95%CI)					
	Model 1		Model 2		Model 3	
	OR (95%CI)	*P* value	OR (95%CI)	*P* value	OR (95%CI)	*P* value
Postmenopausal						
Per-SD increase	1.39(1.21,1.69)	<0.001	1.40(1.25,1.76)	<0.001	1.43(1.26,1.80)	0.002
Quartiles						
Q1(< 0.11)	1.00(Reference)		1.00(Reference)		1.00(Reference)	
Q2(0.11 to 0.22)	1.14 (0.90,1.43)	0.272	1.14 (0.90,1.44)	0.289	1.08 (0.83,1.39)	0.573
Q3(0.22 to 0.31)	1.46 (1.16,1.86)	0.002	1.48 (1.15,1.89)	0.002	1.48 (1.14,1.94)	0.004
Q4(≥0.31)	1.68 (1.32,2.13)	<0.001	1.75 (1.37,2.25)	<0.001	1.58 (1.20,2.07)	0.001
*P* for trend		<0.001		<0.001		<0.001
Premenopausal						
Per-SD increase	1.28(1.11,1.49)	0.013	1.15(1.04,1.41)	0.047	1.01(0.89,1.13)	0.112
Quartiles						
Q1(< 0.11)	1.00(Reference)		1.00(Reference)		1.00(Reference)	
Q2(0.11 to 0.22)	1.11 (0.78,1.59)	0.56	1.09 (0.76,1.57)	0.637	0.97 (0.66,1.45)	0.895
Q3(0.22 to 0.31)	1.29 (0.90,1.86)	0.166	1.27 (0.87,1.84)	0.214	1.12 (0.75,1.67)	0.588
Q4(≥0.31)	1.79 (1.23,2.60)	0.002	1.83 (1.24,2.69)	0.002	1.38 (1.07,1.72)	0.008
*P* for trend		0.017		0.044		0.049
*P* value for interaction		0.001		0.013		0.051

OR, Odds Ratio; CI, Confidence Interval.

Model 1: was adjusted for none.

Model 2: was adjusted for age, education, diabetes, current smoking, alcohol intake and BMI.

Model 3: was adjusted for age, education, diabetes, current smoking, alcohol intake, BMI, LDL_C, TC, blood glucose, HbA1c, the use of antihypertensive drugs and blood-lipid lowering drugs.

Given the influence of lipid-related indicators, particularly triglycerides and HDL, on the atherogenic index of plasma, we analyzed lipid and glucose variables from 2011 as independent predictors and blood pressure values from 2015 as the outcome to construct receiver operating characteristic (ROC) curves. The area under the curve (AUC) was used to assess predictive accuracy (25). The ROC curve analysis revealed that AIP (AUC=0.6051, 95% CI:0.5912 to 0.6190, *P*<0.001), BMI (AUC=0.5801, 95% CI: 0.5658 to 0.5944, *P*<0.001), TG (AUC=0.5750, 95% CI:0.5596 to 0.5903, *P*<0.001), HDL-C (AUC=0.5652, 95% CI: 0.5463 to 0.5841, *P*<0.001), and blood glucose (AUC=0.5643, 95% CI:0.5487 to 0.5799, *P*<0.001) all had significant impacts on the incidence of pre-hypertension and hypertension in the middle-aged and elderly population.

**Figure 9:**
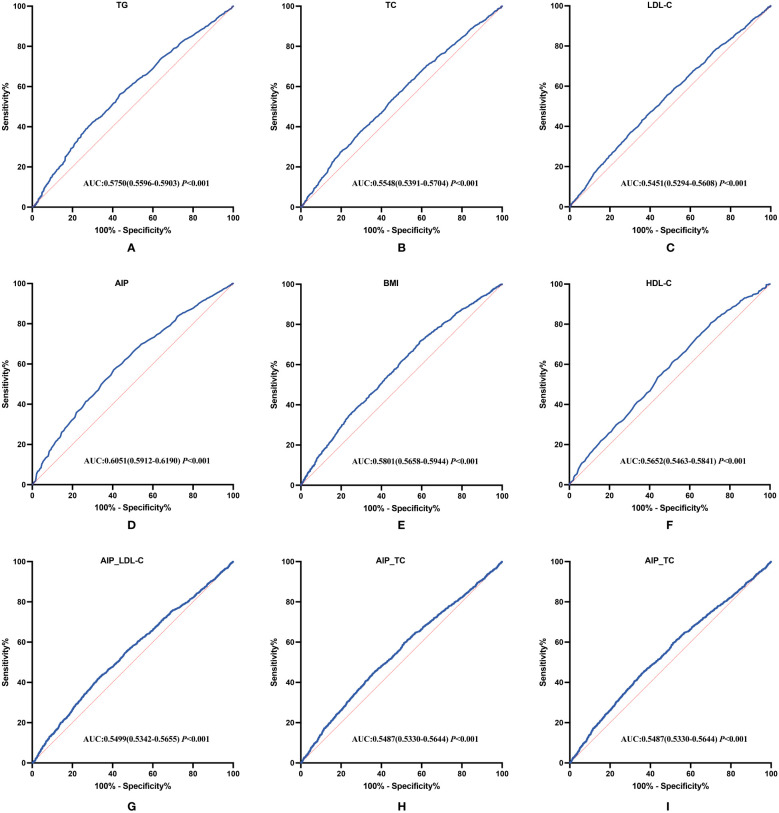
ROC curve analysis of hematological parameters for predicting pre-hypertension and hypertension in the middle-aged and elderly Chinese population. **(A–I)** ROC curve of TG, TC, LDL-C, AIP, BMI, HDL-C, AIP_LDL-C, AIP_TC, AIP_BMI.

It is noteworthy that combined models including AIP_BMI, AIP_LDL-C, and AIP_TC also demonstrated modest predictive value, with results as follows: AIP_BMI (AUC=0.5498, 95% CI:0.5354 to 0.5643, *P*<0.001), AIP_LDL-C (AUC=0.5499, 95% CI:0.5342 to 0.5655, *P*<0.001), and AIP_TC (AUC=0.5487, 95% CI:0.5330 to 0.5644, *P*<0.001). Importantly, the AIP, calculated using HDL-C and TG, exhibited significantly greater predictive value compared to other lipid-related indicators and combined models (**Figure 9**).

## 4 Discussion

This study aimed to investigate the association between AIP exposure and the risk of developing pre-hypertension and hypertension. Individuals with higher AIP exposure exhibited a significantly elevated risk of developing both pre-hypertension and hypertension compared to those with lower AIP levels, a trend particularly pronounced in postmenopausal women. Our findings further demonstrated that AIP serves as a more accurate predictor of pre-hypertension and hypertension risk in menopausal women than other lipid-related parameters (11, 26).

Moreover, pertinent evidence also suggests that the AIP index similarly influences the risk of cardiovascular disease occurrence and overall mortality, as reported in studies (27, 28). With the continuous improvement in living standards and the gradual acceleration of global aging, the population suffering from dyslipidemia is steadily growing. Among these individuals, atherosclerosis is becoming increasingly prevalent, and the AIP index, which is associated with atherosclerosis, has garnered significant attention in recent studies (28).

Our results confirmed a significant, nonlinear relationship between the AIP index and both pre-hypertension and hypertension among individuals, with notable sex-specific differences. When compared to the group with the lowest AIP values, the highest AIP group showed a 69% increased risk of developing pre-hypertension and hypertension (OR: 1.69, 95% CI: 1.38 to 2.07, *P*<0.001). Further subgroup analysis unveiled that the women in the high-AIP group exhibited a 79% increased risk (OR: 1.79, 95% CI: 1.35 to 2.38, *P*<0.001), whereas men showed only a 30% increase (OR: 1.30, 95% CI: 1.12 to 1.79, *P*=0.006). The *P* value for interaction was less than 0.001.

A study conducted by researchers in Gifu Prefecture, Japan, has revealed a significant positive correlation between higher AIP values and the risk of developing pre-hypertension or hypertension. This trend was particularly evident among female participants in the highest quartile of AIP (Q4), with an odds ratio (OR) of 2.19 and a *P*-value of 0.001. Notably, the middle-aged group, approximately 50 years old, exhibited the most pronounced association, with an OR of 2.20 and a *P*-value of 0.007 (29), this study suggests that, due to changes in physiological cycles and hormones, the mechanisms underlying dyslipidemia and hypertension may differ between women and men. In women, menopause-induced estrogen deficiency may lead to metabolic disruptions, commonly manifesting as decreased HDL-C levels and increased LDL-C and TG levels during the perimenopausal period.

The existing literature indicates that, in middle-aged and elderly women, the reduction and depletion of endogenous ovarian hormones following menopause is a primary factor contributing to the heightened risk of visceral obesity (30). Similarly, Gurka, Matthew J. et al. (31) discovered that postmenopausal women exhibit a higher prevalence of metabolic syndrome and an elevated risk of cardiovascular disease. Ben Ali Samir et al. (32) compared the incidence of hypertension between premenopausal and postmenopausal women and found that the latter group had a significantly higher rate of hypertension. The findings of this study emphasize the significance of considering sex as a modifying factor when assessing the impact of the AIP on blood pressure conditions, particularly in the context of postmenopausal women’s health. The observed disparities underscore the necessity for tailored interventions and further investigation into the underlying mechanisms linking AIP, sex, and hypertension risk.

Our stratified analysis revealed an intriguing phenomenon: postmenopausal women exhibited significantly higher lipid profiles and elevated risks of pre-hypertension and hypertension compared to non-menopausal counterparts. With prolonged follow-up time, the population with a high AIP exhibited a significantly elevated risk of developing prehypertension and hypertension. In the female cohort, the association between high AIP and the risk of prehypertension/hypertension increased from 1.49-fold (OR=1.49, 95% CI:1.14 to 1.95, *P*=0.003) in 2015 to 1.64-fold (OR=1.64, 95% CI:1.31 to 2.06, *P*<0.001) in 2018, with this trend being particularly pronounced among postmenopausal women. We hypothesize that this association may be closely linked to the dramatic decline in endogenous estrogen levels following menopause. Multiple studies have suggested that estrogen regulates lipid metabolism balance through multifaceted mechanisms. In terms of lipid component modulation, estrogen elevates HDL-C levels by promoting its synthesis and inhibiting its breakdown in the liver, while accelerating LDL-C clearance via upregulating hepatic LDL receptor expression. It also maintains triglyceride balance by suppressing hepatic triglyceride synthase activity (33, 34). Postmenopausal estrogen decline leads to weakened HDL-C metabolism, reduced LDL-C clearance efficiency, and increased triglyceride accumulation risks. Regarding fat distribution, estrogen drives region-specific fat deposition in areas like the hips and thighs, whereas estrogen deficiency correlates with visceral fat accumulation and diminished adipocyte lipolysis efficiency. Metabolically, estrogen exerts cardiovascular protection by lowering TC and LDL-C while enhancing cholesterol reverse transport. However, postmenopausal increases in the LDL-C/HDL-C ratio combined with chronic inflammatory factor release exacerbate lipid dysregulation, significantly raising risks of hypertriglyceridemia and insulin resistance. Molecularly, estrogen activates nuclear receptors to regulate key lipid-metabolizing enzymes (such as LPL and HL) and inhibits the NF-κB pathway to mitigate inflammatory interference in lipid metabolism. These mechanisms collectively highlight the dynamic interplay between estrogen levels and lipid homeostasis, underscoring estrogen’s pivotal role in menopausal metabolic syndrome development (33, 35, 36).

The AIP index, derived from a combination of TG and HDL-C results, demonstrates a certain predictive value for the onset of pre-hypertension and hypertension, as evidenced by the ROC curve analysis. Both when considered individually (TG or HDL-C) and as the AIP index, they exhibit an AUC value greater than 0.56. Numerous studies have confirmed a close association between elevated levels of TG and altered HDL-C with insulin resistance, suggesting that their ratio may serve as a surrogate marker for this condition (37, 38). These lipids impair insulin’s ability to bind with glucose by inhibiting the activity of insulin receptors on fat cells. Additionally, an increase in HDL-C levels can affect insulin secretion and reduce its sensitivity, ultimately leading to elevated blood glucose levels (11, 39–41). Insulin resistance, an independent high-risk factor for hypertension, exhibits a complex mechanism that significantly impacts blood pressure regulation (42). Elevated insulin levels promote sodium reabsorption in the proximal renal tubules, resulting in water and sodium retention, which increases blood volume and sustains elevated blood pressure. Furthermore, insulin resistance stimulates the sympathetic nervous system, increasing the concentrations of excitatory neurotransmitters such as catecholamines, thereby exacerbating vascular constriction, increasing peripheral resistance, and contributing to the development of hypertension. Lastly, persistent high insulin levels may disrupt normal endothelial function, inhibiting the production of vasodilators and promoting the production of vasoconstrictors (such as endothelin-1), thus disturbing the balance between vasodilation and vasoconstriction (42, 43).

The prospective longitudinal study conducted by Lin Chia-Hung et al. (44) has further confirmed the close relationship between the progression of hypertension and insulin resistance. Insulin resistance not only plays a pivotal role in the development of cognitive impairment among elderly patients with primary hypertension but also stands as an independent risk factor for its advancement (38). Notably, the primary study index included in this research, the AIP, can largely mirror the blood lipid levels of the study population, thereby indirectly reflecting the intricate association between dyslipidemia, insulin resistance, and hypertension. This discovery aligns with the findings of numerous studies and offers a fresh perspective on understanding the pathogenesis of cardiovascular diseases.

A high AIP index is frequently associated with elevated lipid levels and obesity. Excess weight can induce alterations in adiponectin secretion, resulting in an increase in free fatty acid levels and a decrease in adiponectin levels. These changes, in turn, can impair microvascular function to varying degrees, leading to capillary shedding, arteriolar constriction, and heightened peripheral resistance, ultimately elevating blood pressure (45). Furthermore, in obese individuals, the regulatory mechanism of vascular reactivity often shifts towards vasoconstriction, enhancing the effects of endothelin (a vasoconstrictor) while diminishing the effects of nitric oxide (a vasodilator). Additionally, communication pathways between adipose tissue and the vascular system, including sympathetic nervous activity and the release of adiponectin, are significantly intensified. All these factors contribute to microvascular dysfunction in the body, exacerbating the elevation of blood pressure (46). Our research findings align with these observations, indicating that middle-aged and older women are more significantly affected by the AIP index in terms of blood pressure levels compared to men. Specifically, women with a high AIP index face a notably higher risk of developing pre-hypertension and progressing to hypertension than their male counterparts in the same age group.

The findings of this study suggest that an elevated atherogenic index of plasma (AIP) may serve as a predictive marker for hypertension risk in middle-aged and elderly women, particularly in perimenopausal and postmenopausal populations. This discovery highlights the potential value of early monitoring and intervention strategies for individuals with high AIP levels, which could help reduce the progression rate from prehypertension to overt hypertension and delay the clinical progression of the disease. These insights may contribute to the scientific basis for primary prevention of cardiovascular diseases. Further validation of its predictive efficacy and exploration of AIP-based risk-stratified management models could not only optimize personalized health interventions but also reduce hypertension-related disease burdens, thereby demonstrating potential public health significance and clinical translational relevance.

While this study provides valuable insights, it is crucial to acknowledge its limitations. (1) The study population exclusively comprised Chinese individuals, necessitating caution when generalizing the findings to other ethnic groups or populations from different countries. (2) The cohort predominantly focused on middle-aged and older adults, whereas hypertension prevalence is demonstrating an increasingly younger-onset trend. Therefore, extending these findings to younger age groups may require further validation. (3) Although the study adjusted for known confounders, potential residual confounding factors, such as dietary habits, exercise patterns, and activity frequency, were not fully accounted for and might influence the outcomes. (4) As the research relied on questionnaire-based data, biases from unmeasurable or unrecognized factors cannot be entirely eliminated. (5) Due to the observational nature of this study, even with the inclusion of a longitudinal cohort design, direct causal inferences between AIP levels and the risks of pre-hypertension and hypertension remain constrained. Further prospective studies or interventional trials are essential to definitively establish the causal relationship between these variables.

## 5 Conclusion

This study, focusing on middle-aged and older adults in China, revealed that elevated AIP levels exhibited significantly positive associations with both the incidence of pre-hypertension and hypertension. The risk of these conditions progressively increased with rising AIP values, with the most pronounced effects observed in postmenopausal women. Therefore, regular monitoring and maintaining optimal AIP levels play a critical role in preventing hypertension onset and potentially delaying or halting the progression from pre-hypertension to hypertension. In the future, AIP has the potential to serve as a biomarker for hypertension risk surveillance, though further large-scale prospective studies are warranted to validate this hypothesis.

## Data Availability

The original contributions presented in the study are included in the article/**Supplementary Material**. Further inquiries can be directed to the corresponding author.

## References

[1] Zhang M Shi Y Zhou B Huang Z Zhao Z Li CB. Prevalence, awareness, treatment, and control of hypertension in China, 2004-18: findings from six rounds of a national survey. BMJ. (2023) 380:e071952. doi: 10.1136/bmj-2022-071952 36631148 PMC10498511

[2] Nguyen TN Chow CK Global and national high blood pressure burden and control. Lancet. (2021) 398:932–3. doi: 10.1016/S0140-6736(21)01688-3 34450082

[3] Worldwide trends in hypertension prevalence and progress in treatment and control from 1990 to 2019: a pooled analysis of 1201 population-representative studies with 104 million participants. Lancet. (2021) 398:957–80. doi: 10.1016/S0140-6736(21)01330-1; PMC8446938 34450083

[4] Zhou M Wang H Zeng X Yin P Zhu J Chen W. Mortality, morbidity, and risk factors in China and its provinces, 1990-2017: a systematic analysis for the Global Burden of Disease Study 2017. Lancet. (2019) 394:1145–58. doi: 10.1016/S0140-6736(19)30427-1 PMC6891889 31248666

[5] Ventura HO Lavie CJ Antihypertensive therapy for prehypertension: relationship with cardiovascular outcomes. Jama-j Am Med Assoc. (2011) 305:940–1. doi: 10.1001/jama.2011.256 21364146

[6] DeCarli C Blood pressure control and cognitive performance: something to think about with aging. JAMA. (2015) 313:1963–4. doi: 10.1001/jama.2015.3113 PMC5540364 25988465

[7] Lee SS Ae Kong K Kim D Lim YM Yang PS Yi JED. Clinical implication of an impaired fasting glucose and prehypertension related to new onset atrial fibrillation in a healthy Asian population without underlying disease: a nationwide cohort study in Korea. Eur Heart J. (2017) 38:2599–607. doi: 10.1093/eurheartj/ehx316 28662568

[8] Liu Z Zhang L Wang L Li K Fan F Jia J. The predictive value of cumulative atherogenic index of plasma (AIP) for cardiovascular outcomes: a prospective community-based cohort study. Cardiovasc Diabetol. (2024) 23:264. doi: 10.1186/s12933-024-02350-8 39026310 PMC11264486

[9] Fu L Zhou Y Sun J Zhu Z Xing Z Zhou S. Atherogenic index of plasma is associated with major adverse cardiovascular events in patients with type 2 diabetes mellitus. Cardiovasc Diabetol. (2021) 20:201. doi: 10.1186/s12933-021-01393-5 34610830 PMC8493717

[10] Zou Y Lu S Li D Huang X Wang C Xie G. Exposure of cumulative atherogenic index of plasma and the development of prediabetes in middle-aged and elderly individuals: evidence from the CHARLS cohort study. Cardiovasc Diabetol. (2024) 23:355. doi: 10.1186/s12933-024-02449-y 39350154 PMC11443941

[11] Qin M Chen B Association of atherogenic index of plasma with cardiovascular disease mortality and all-cause mortality in the general US adult population: results from NHANES 2005-2018. Cardiovasc Diabetol. (2024) 23:255. doi: 10.1186/s12933-024-02359-z 39014415 PMC11253368

[12] Zheng Y Li C Yang J Seery S Qi Y Wang W. Atherogenic index of plasma for non-diabetic, coronary artery disease patients after percutaneous coronary intervention: a prospective study of the long-term outcomes in China. Cardiovasc Diabetol. (2022) 21:29. doi: 10.1186/s12933-022-01459-y 35193553 PMC8864872

[13] Barua L Faruque M Banik PC Ali L Atherogenic index of plasma and its association with cardiovascular disease risk factors among postmenopausal rural women of Bangladesh. Indian Heart J. (2019) 71:155–60. doi: 10.1016/j.ihj.2019.04.012 PMC6620423 31280829

[14] Guo Q Zhou S Feng X Yang J Qiao J Zhao Y. The sensibility of the new blood lipid indicator–atherogenic index of plasma (AIP) in menopausal women with coronary artery disease. Lipids Health Dis. (2020) 19:27. doi: 10.1186/s12944-020-01208-8 32093690 PMC7041294

[15] Moradi L Hashemi SJ Zaman F Alipour M Farhangiyan Z Sharifzadeh M. Comparison of metabolic risk factors, lipid indices, healthy eating index, and physical activity among premenopausal, menopausal, and postmenopausal women. Rom J Intern Med. (2024) 62:260–71. doi: 10.2478/rjim-2024-0012 38536781

[16] Elmugadam A Elfadil GA Hamad AI El Shikieri AB Aledrissy M Altayb HN. Atherogenic index of plasma and anthropometric measurements among osteoporotic postmenopausal Sudanese women: possible risk for cardiovascular disease. J Aging Res. (2022) 2022:1545127. doi: 10.1155/2022/1545127 36199371 PMC9529371

[17] Gong J Wang G Wang Y Chen X Chen Y Meng Q. Nowcasting and forecasting the care needs of the older population in China: analysis of data from the China Health and Retirement Longitudinal Study (CHARLS). Lancet Public Health. (2022) 7:e1005–e13. doi: 10.1016/S2468-2667(22)00203-1 PMC9741660 36423656

[18] Zhao Y Hu Y Smith JP Strauss J Yang G Cohort profile: the China health and retirement longitudinal study (CHARLS). Int J Epidemiol. (2014) 43:61–8. doi: 10.1093/ije/dys203 PMC3937970 23243115

[19] Onat A Can G Kaya H Hergenç G Atherogenic index of plasma” (log10 triglyceride/high-density lipoprotein-cholesterol) predicts high blood pressure, diabetes, and vascular events. J Clin Lipidol. (2010) 4:89–98. doi: 10.1016/j.jacl.2010.02.005 21122635

[20] 2018 chinese guidelines for prevention and treatment of hypertension-A report of the revision committee of chinese guidelines for prevention and treatment of hypertension. J Geriatr Cardiol. (2019) 16:182–241. doi: 10.11909/j.issn.1671-5411.2019.03.014 31080465 PMC6500570

[21] Zhang L Li JL Guo LL Li H Li D Xu G. The interaction between serum uric acid and triglycerides level on blood pressure in middle-aged and elderly individuals in China: result from a large national cohort study. BMC Cardiovasc Disord. (2020) 20:174. doi: 10.1186/s12872-020-01468-3 32293295 PMC7160924

[22] Luo X Yang H He Z Wang S Li C Chen T. Numbers and mortality risk of hypertensive patients with or without elevated body mass index in China. Int J Environ Res Public Health. (2021) 19(1). doi: 10.3390/ijerph19010116 PMC8750230 35010380

[23] Chen X Crimmins E Hu PP Kim JK Meng Q Strauss J. Venous blood-based biomarkers in the China health and retirement longitudinal study: rationale, design, and results from the 2015 wave. Am J Epidemiol. (2019) 188:1871–7. doi: 10.1093/aje/kwz170 PMC6825825 31364691

[24] Wu Y Yang Y Zhang J Liu S Zhuang W The change of triglyceride-glucose index may predict incidence of stroke in the general population over 45 years old. Cardiovasc Diabetol. (2023) 22:132. doi: 10.1186/s12933-023-01870-z 37296457 PMC10257314

[25] Xu J Qu P Du X Xiang Q Guo L Zhu L. Change in postprandial level of remnant cholesterol after a daily breakfast in chinese patients with hypertension. Front Cardiovasc Med. (2021) 8:685385. doi: 10.3389/fcvm.2021.685385 34212015 PMC8239280

[26] Shi Y Wen M Sex-specific differences in the effect of the atherogenic index of plasma on prediabetes and diabetes in the NHANES 2011-2018 population. Cardiovasc Diabetol. (2023) 22:19. doi: 10.1186/s12933-023-01740-8 36717829 PMC9887826

[27] Min Q Wu Z Yao J Wang S Duan L Liu S. Association between atherogenic index of plasma control level and incident cardiovascular disease in middle-aged and elderly Chinese individuals with abnormal glucose metabolism. Cardiovasc Diabetol. (2024) 23:54. doi: 10.1186/s12933-024-02144-y 38331798 PMC10854096

[28] Duiyimuhan G Maimaiti N The association between atherogenic index of plasma and all-cause mortality and cardiovascular disease-specific mortality in hypertension patients: a retrospective cohort study of NHANES. BMC Cardiovasc Disord. (2023) 23:452. doi: 10.1186/s12872-023-03451-0 37697281 PMC10496369

[29] Tan M Zhang Y Jin L Wang Y Cui W Nasifu L. Association between atherogenic index of plasma and prehypertension or hypertension among normoglycemia subjects in a Japan population: a cross-sectional study. Lipids Health Dis. (2023) 22:87. doi: 10.1186/s12944-023-01853-9 37386459 PMC10308786

[30] Meyer MR Clegg DJ Prossnitz ER Barton M Obesity, insulin resistance and diabetes: sex differences and role of oestrogen receptors. Acta Physiol. (2011) 203:259–69. doi: 10.1111/j.1748-1716.2010.02237.x PMC3110567 21281456

[31] Gurka MJ Vishnu A Santen RJ DeBoer MD Progression of metabolic syndrome severity during the menopausal transition. J Am Heart Assoc. (2016) 5(8). doi: 10.1161/JAHA.116.003609 PMC5015287 27487829

[32] Ben Ali S Belfki-Benali H Ahmed DB Haddad N Jmal A Abdennebi M. Postmenopausal hypertension, abdominal obesity, apolipoprotein and insulin resistance. Clin Exp Hypertens. (2016) 38:370–4. doi: 10.3109/10641963.2015.1131286 27149156

[33] Khakurel G Kayastha R Chalise S Karki PK Atherogenic index of plasma in postmenopausal women. J Nepal Health Res Counc. (2018) 16:175–7. doi: 10.33314/jnhrc.v16i2.1570 29983433

[34] Viktorinova A Brnka R Pirosova M Pontuch P Kinova S Sex differences in the correlation between lipids related to cardiovascular risk factors and small dense LDL particles in patients with type 2 diabetes. Arch Endocrin Metab. (2024) 68:e240069. doi: 10.20945/2359-4292-2024-0069 PMC11460969 39420941

[35] Bajerska J Skoczek-Rubińska A Małczak L Vucic V Arsic A Kojadinovic M. Plasma fatty acid composition and some markers of dietary habits are associated with cardiovascular disease risk determined by an atherogenic plasma index in postmenopausal women. Nutr Res. (2023) 115:47–60. doi: 10.1016/j.nutres.2023.05.008 37300953

[36] Wu TT Gao Y Zheng YY Ma YT Xie X Atherogenic index of plasma (AIP): a novel predictive indicator for the coronary artery disease in postmenopausal women. Lipids Health Dis. (2018) 17:197. doi: 10.1186/s12944-018-0828-z 30134981 PMC6106932

[37] Oliveri A Rebernick RJ Kuppa A Pant A Chen Y Du X. Comprehensive genetic study of the insulin resistance marker TG: HDL-C in the UK Biobank. Nat Genet. (2024) 56:212–21. doi: 10.1038/s41588-023-01625-2 PMC10923176 38200128

[38] Ma L Feng M Qian Y Yang W Liu J Han R. Insulin resistance is an important risk factor for cognitive impairment in elderly patients with primary hypertension. Yonsei Med J. (2015) 56:89–94. doi: 10.3349/ymj.2015.56.1.89 25510751 PMC4276782

[39] Goodpaster BH Kelley DE Skeletal muscle triglyceride: marker or mediator of obesity-induced insulin resistance in type 2 diabetes mellitus? Curr Diabetes Rep. (2002) 2:216–22. doi: 10.1007/s11892-002-0086-2 12643176

[40] Chu NF Spiegelman D Hotamisligil GS Rifai N Stampfer M Rimm EB. Plasma insulin, leptin, and soluble TNF receptors levels in relation to obesity-related atherogenic and thrombogenic cardiovascular disease risk factors among men. Atherosclerosis. (2001) 157:495–503. doi: 10.1016/S0021-9150(00)00755-3 11472752

[41] Lin D Qi Y Huang C Wu M Wang C Li F. Associations of lipid parameters with insulin resistance and diabetes: A population-based study. Clin Nutr. (2018) 37:1423–9. doi: 10.1016/j.clnu.2017.06.018 28673690

[42] Ferrannini E Cushman WC Diabetes and hypertension: the bad companions. Lancet. (2012) 380:601–10. doi: 10.1016/S0140-6736(12)60987-8 22883509

[43] Zhi H Wang H Li T Pin F Correlated analysis and pathological study on insulin resistance and cardiovascular endocrine hormone in elderly hypertension patients. Diabetes Metab synd. (2015) 9:67–70. doi: 10.1016/j.dsx.2015.02.013 25796973

[44] Lin CH Wei JN Fan KC Fang CT Wu WC Yang CY. Different cutoffs of hypertension, risk of incident diabetes and progression of insulin resistance: A prospective cohort study. J formos Med Assoc. (2022) 121:193–201. doi: 10.1016/j.jfma.2021.02.022 33766449

[45] Karaca Ü Schram MT Houben AJ Muris DM Stehouwer CD Microvascular dysfunction as a link between obesity, insulin resistance and hypertension. Diabetes Res Clin Pr. (2014) 103:382–7. doi: 10.1016/j.diabres.2013.12.012 24438874

[46] de Jongh RT Serné EH Eringa EC RG IJ Stehouwer CD Does microvascular dysfunction link obesity with insulin resistance and hypertension? Expert Rev Endocrino. (2006) 1:181–7. doi: 10.1586/17446651.1.2.181 30754148

