# Supplementary material for: Sex-specific differences in the relationship between the atherogenic index and hypertension in middle-aged and elderly Chinese
Source: Front Endocrinol (Lausanne). 2025 Jun 18;16:1574125. doi: 10.3389/fendo.2025.1574125 (PMC12213374; doi:10.3389/fendo.2025.1574125)
Supplement: Supplementary file 4 [file Supplementaryfile1.docx]

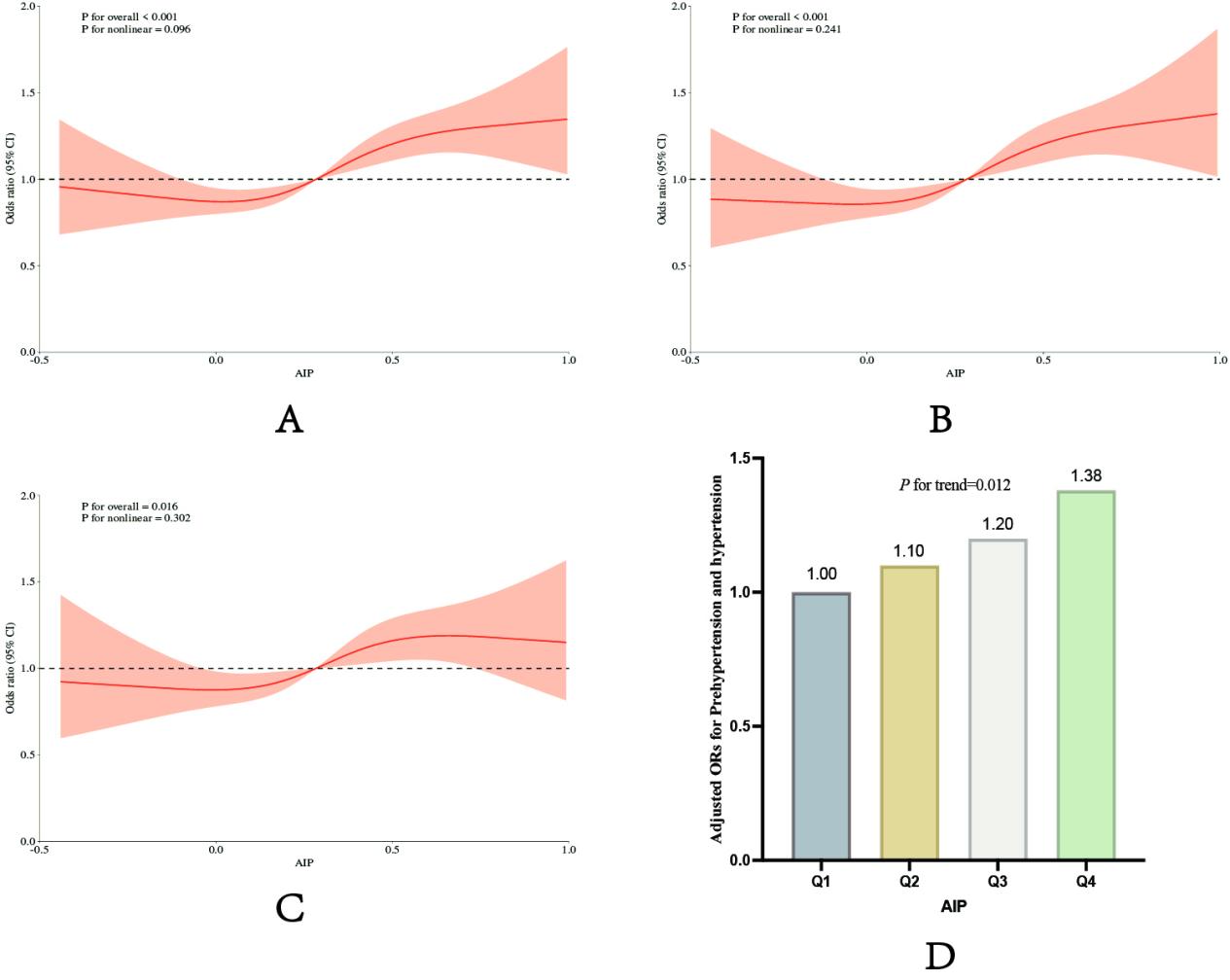


Figure S1


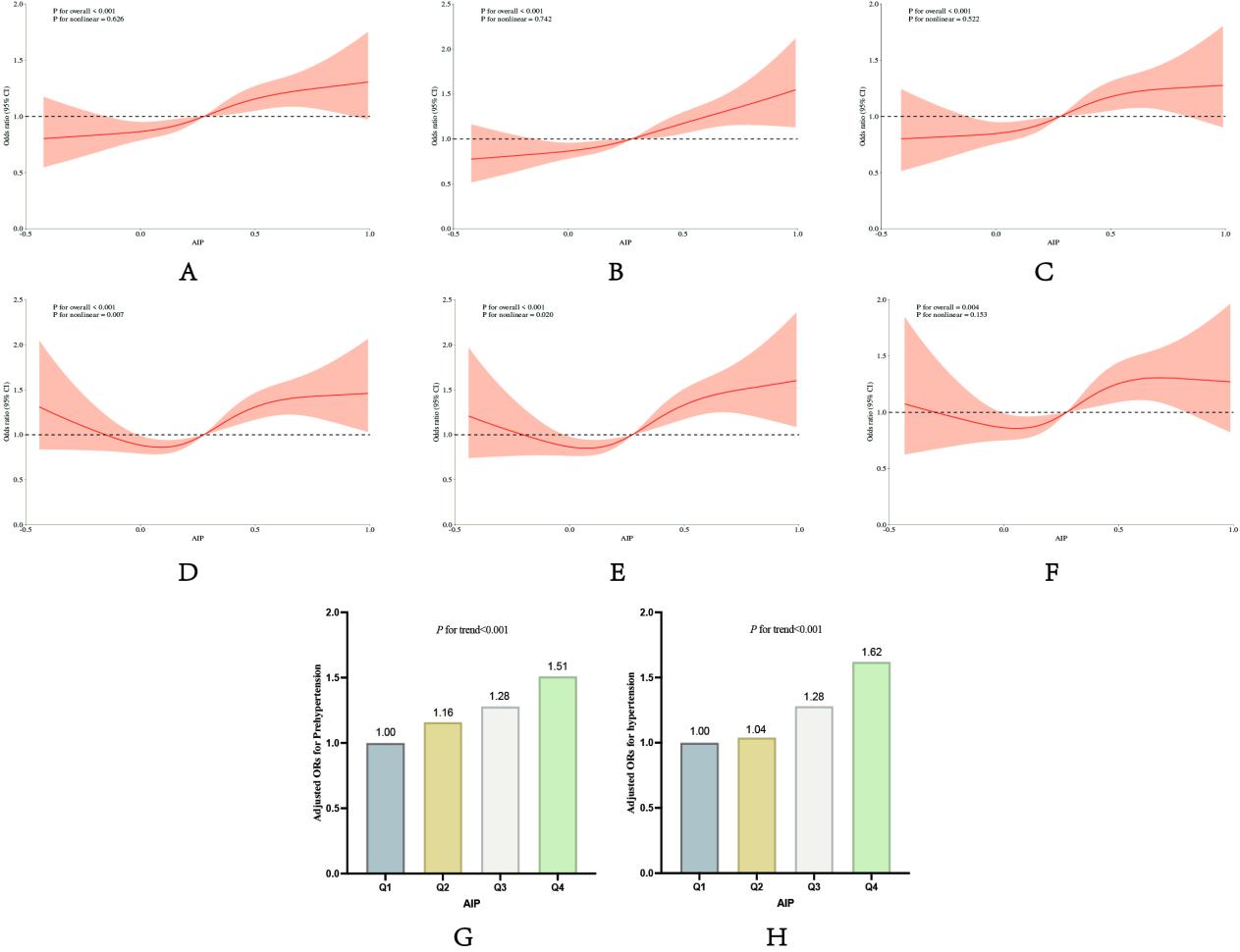


Figure S2


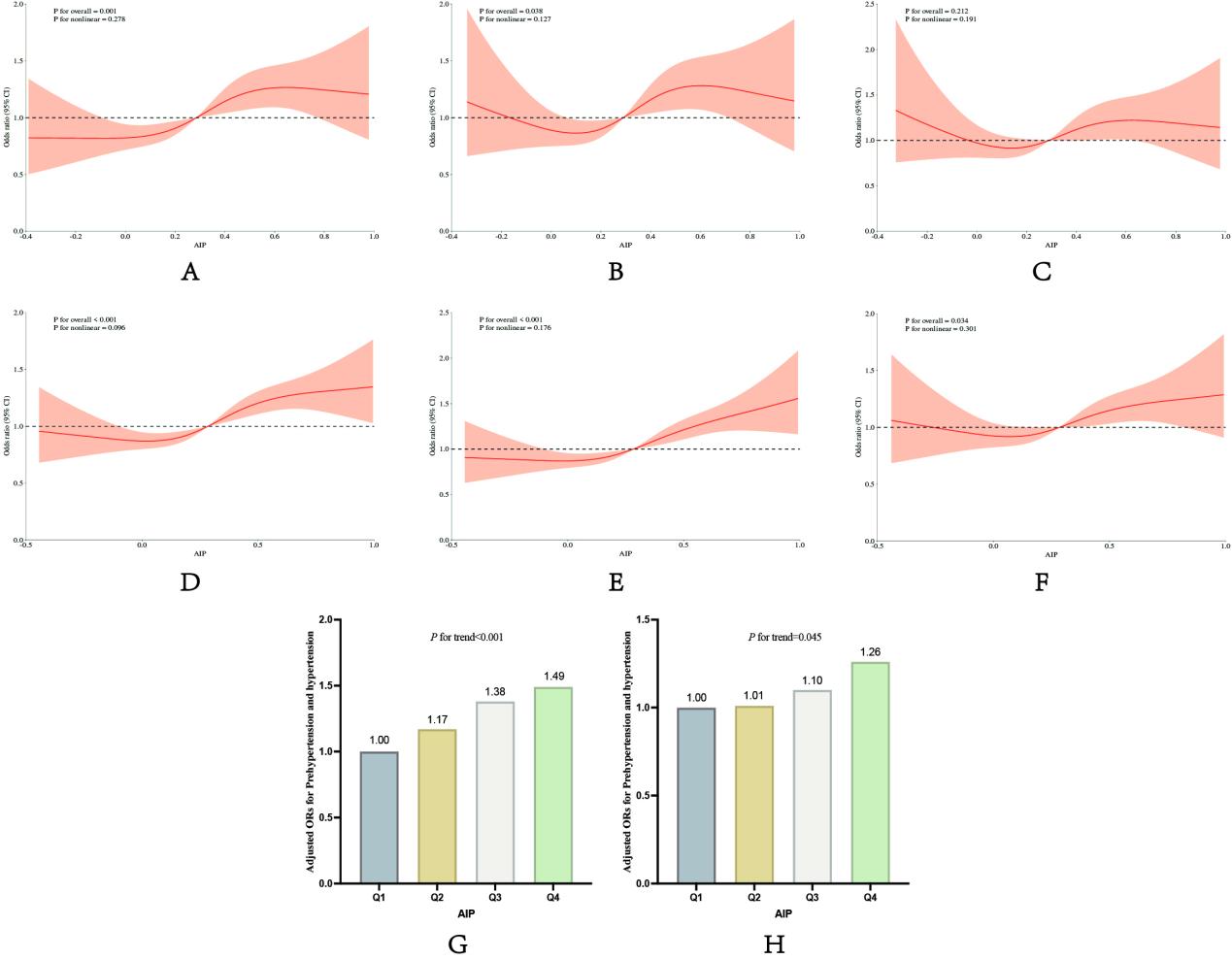


Figure S3

**Figure 8** AIP and Hypertension Prevalence by Menopausal Status: 2018 Subgroup Analysis‌. (A)RCS curves of unadjusted AIP and prehypertension/hypertension prevalence in premenopausal women, (B)RCS curves of AIP and pre-hypertension/hypertension incidence after adjusting for covariates such as age, sex, education, age, smoking and drinking status, and BMI in premenopausal women, (C)RCS curves of AIP and pre-hypertension/hypertension incidence after adjusting for covariates such as LDL_C, TC, blood glucose, HbA1c, the use of antihypertensive drugs and blood-lipid lowering drugs based on (B), (D) RCS curves of unadjusted AIP and prehypertension/hypertension prevalence in postmenopausal women, (E)RCS curves of AIP and pre-hypertension/hypertension incidence after adjusting for covariates such as age, sex, education, age, smoking and drinking status, and BMI in women, (F) RCS curves of AIP and pre-hypertension/hypertension incidence after adjusting for covariates such as LDL_C, TC, blood glucose, HbA1c, the use of antihypertensive drugs and blood-lipid lowering drugs based on (E), (G) Relative odds of pre-hypertension/hypertension corresponding to quartiles of AIP.
