# Supplementary material for: Sex-specific differences in the relationship between the atherogenic index and hypertension in middle-aged and elderly Chinese
Source: Front Endocrinol (Lausanne). 2025 Jun 18;16:1574125. doi: 10.3389/fendo.2025.1574125 (PMC12213374; doi:10.3389/fendo.2025.1574125)
Supplement: Supplementary file 5 [file Table1.docx]

| **Table S1 The relative risks of pre-hypertension and hypertension in all subjects were calculated according to different AIP models in longitudinal study of 2015** | | | | | | | | |
| --- | --- | --- | --- | --- | --- | --- | --- | --- |
| AIP | Pre-hypertension and hypertension OR (95%CI) | | | | | | | |
|  | Model 1 | |  | Model 2 | |  | Model 3 | |
|  | OR (95%CI) | *P* value |  | OR (95%CI) | P value |  | OR (95%CI) | *P* value |
| Per-SD increase | 1.67(1.41,1.80) | <0.001 |  | 1.54(1.33,1.67) | 0.009 |  | 1.41(1.31,1.53) | 0.014 |
| Quartiles |  |  |  |  |  |  |  |  |
| Q1(< 0.11) | 1.00(Reference) |  |  | 1.00（Reference) |  |  | 1.00（Reference) |  |
| Q2(0.22 to 0.24) | 1.05 (0.92,1.20) | 0.48 |  | 1.08 (0.93, 1.25) | 0.298 |  | 1.10 (0.93 ,1.30) | 0.257 |
| Q3(0.24 to 0.30) | 1.21 (1.05,1.39) | 0.008 |  | 1.20 (1.03,1.40) | 0.016 |  | 1.20 (1.01,1.43) | 0.045 |
| Q4(≥ 0.30) | 1.47 (1.28,1.70) | <0.001 |  | 1.52 (1.30, 1.78) | <0.001 |  | 1.38 (1.13,1.67) | 0.001 |
| *P* for trend |  | 0.003 |  |  | 0.007 |  |  | 0.012 |
| OR: Odds Ratio, CI: Confidence Interval | | | | | | | | |
| Model1: was adjusted for none | | | | | | | | |
| Model2: was adjusted for age, sex, education, diabetes, current smoking, alcohol intake, and BMI | | | | | | | | |
| Model3: was adjusted for age, sex, education, diabetes, current smoking, alcohol intake, BMI, LDL_C, TC, blood glucose, HbA1c， the use of antihypertensive drugs, and blood-lipid lowering drugs | | | | | | | | |
|  |  |  |  |  |  |  |  |  |

| **Table S2 Multivariate logistic regression analysis of the relationship between AIP and pre-hypertension as well as hypertension in Longitudinal study in 2015** | | | | | | | | |
| --- | --- | --- | --- | --- | --- | --- | --- | --- |
| AIP | Model 1 | |  | Model 2 | |  | Model 3 | |
|  | OR (95%CI) | *P* value |  | OR (95%CI) | *P* value |  | OR (95%CI) | *P* value |
| Pre-hypertension |  |  |  |  |  |  |  |  |
| Per-SD increase | 1.47(1.21,1.66) | 0.014 |  | 1.61(1.33,1.89) | 0.006 |  | 1.55(1.22,2.01) | <0.001 |
| Quartiles |  |  |  |  |  |  |  |  |
| Q1(< 0.11) | 1.00(Reference) |  |  | 1.00(Reference) |  |  | 1.00(Reference) |  |
| Q2(0.22 to 0.24) | 1.08 (0.92,1.25) | 0.351 |  | 1.11 (0.94,1.31) | 0.218 |  | 1.16 (0.97,1.39) | 0.112 |
| Q3(0.24 to 0.30) | 1.19 (1.01,1.39) | 0.033 |  | 1.21 (1.02,1.44) | 0.026 |  | 1.28 (1.06,1.54) | 0.009 |
| Q4(≥ 0.30) | 1.45 (1.23, 1.70) | <0.001 |  | 1.56 (1.31,1.86) | <0.001 |  | 1.51 (1.24,1.83) | <0.001 |
| *P* for trend |  | 0.035 |  |  | 0.019 |  |  | <0.001 |
| Hypertension |  |  |  |  |  |  |  |  |
| Per-SD increase | 1.61(1.44,1.87) | 0.017 |  | 1.67(1.34,1.99) | 0.008 |  | 1.77(1.31,2.07) | 0.001 |
| Quartiles |  |  |  |  |  |  |  |  |
| Q1(< 0.11) | 1.00(Reference) |  |  | 1.00(Reference) |  |  | 1.00(Reference) |  |
| Q2(0.22 to 0.24) | 1.00 (0.83,1.22) | 0.97 |  | 1.05 (0.85,1.30) | 0.622 |  | 1.04 (0.85,1.29) | 0.685 |
| Q3(0.24 to 0.30) | 1.25 (1.03, 1.51) | 0.024 |  | 1.29 (1.04 ,1.60) | 0.02 |  | 1.28 (1.03,1.58) | 0.027 |
| Q4(≥ 0.30) | 1.52 (1.25,1.85) | <0.001 |  | 1.64 (1.32, 2.04) | <0.001 |  | 1.62 (1.30, 2.03) | <0.001 |
| *P* for trend |  | 0.007 |  |  | 0.001 |  |  | <0.001 |
| *P* value for interaction |  | <0.001 |  |  | <0.001 |  |  | <0.001 |
| OR: Odds Ratio, CI: Confidence Interval | | | | | | | | |
| Model1: was adjusted for none | | | | | | | | |
| Model2: was adjusted for age, education, diabetes, current smoking, alcohol intake and BMI | | | | | | | | |
| Model3: was adjusted for age, education, diabetes, current smoking, alcohol intake, BMI, LDL_C, TC, blood glucose, HbA1c, the use of antihypertensive drugs and blood-lipid lowering drugs | | | | | | | | |
|  |  |  |  |  |  |  |  |  |

| **Table S3 Relative risk of pre-hypertension and hypertension in males and females under different AIP models in the longitudinal study of 2015** | | | | | | | | |
| --- | --- | --- | --- | --- | --- | --- | --- | --- |
| AIP | Pre-hypertension and hypertension OR (95%CI) | | | | | | | |
|  | Model 1 | |  | Model 2 | |  | Model 3 | |
|  | OR (95%CI) | *P* value |  | OR (95%CI) | *P* value |  | OR (95%CI) | *P* value |
| Female |  |  |  |  |  |  |  |  |
| Per-SD increase | 1.72(1.33,1.98) | <0.001 |  | 1.58(1.27,2.05) | <0.001 |  | 1.39(1.22,1.69) | 0.014 |
| Quartiles |  |  |  |  |  |  |  |  |
| Q1(< 0.22) | 1.00(Reference) |  |  | 1.00(Reference) |  |  | 1.00(Reference) |  |
| Q2(0.22 to 0.42) | 1.13 (1.03,1.37) | 0.024 |  | 1.12 (1.01,1.38) | 0.035 |  | 1.17 (0.87,1.39) | 0.043 |
| Q3(0.42 to 0.63) | 1.38 (1.13,1.67) | 0.001 |  | 1.34 (1.08,1.65) | 0.007 |  | 1.38 (1.01,1.64) | 0.046 |
| Q4(≥ 0.63) | 1.69 (1.39,2.05) | <.0001 |  | 1.74 (1.40,2.15) | <0.001 |  | 1.49 (1.14,1.95) | 0.003 |
| *P* for trend |  | <0.001 |  |  | <0.001 |  |  | <0.001 |
| Male |  |  |  |  |  |  |  |  |
| Per-SD increase | 1.33(1.09,1.54) | <0.001 |  | 1.21(1.10,1.48) | 0.027 |  | 1.18(1.07,1.33) | 0.049 |
| Quartiles |  |  |  |  |  |  |  |  |
| Q1(< 0.22) | 1.00(Reference) |  |  | 1.00(Reference) |  |  | 1.00(Reference) |  |
| Q2(0.22 to 0.42) | 1.05 (0.92,1.20) | 0.48 |  | 1.09 (0.94, 1.26) | 0.266 |  | 1.01 (0.81, 1.28) | 0.368 |
| Q3(0.42 to 0.63) | 1.21 (1.05, 1.39) | 0.008 |  | 1.22 (1.05,1.42) | 0.01 |  | 1.10 (1.01,1.44) | 0.043 |
| Q4(≥ 0.63) | 1.47 (1.28,1.70) | <0.001 |  | 1.37 (1.14,1.84) | <0.001 |  | 1.26 (1.12,1.66) | 0.002 |
| *P* for trend |  | <0.001 |  |  | 0.014 |  |  | 0.047 |
| *P* value for interaction |  | <0.001 |  |  | 0.006 |  |  | 0.045 |
| OR: Odds Ratio, CI: Confidence Interval | | | | | | | | |
| Model1: was adjusted for none | | | | | | | | |
| Model2: was adjusted for age, education, diabetes, current smoking, alcohol intake and BMI | | | | | | | | |
| Model3: was adjusted for age, education, diabetes, current smoking, alcohol intake, BMI, LDL_C, TC, blood glucose, HbA1c, the use of antihypertensive drugs and blood-lipid lowering drugs | | | | | | | | |
|  |  |  |  |  |  |  |  |  |
